# The safety profile of mesenchymal stem cell therapy administered through intrathecal injections for treating neurological disorders: a systematic review and meta-analysis of randomised controlled trials

**DOI:** 10.1186/s13287-024-03748-7

**Published:** 2024-05-20

**Authors:** Luz Estella Mesa Bedoya, Jhyld Carolaind Camacho Barbosa, Lucas López Quiceno, Freddy Barrios Arroyave, Karolynn Halpert, Julián Andrés España Peña, Juan Carlos Salazar Uribe

**Affiliations:** 1 BioXcellerator/ BioXscience Advanced Therapies and Translational Medicine, Medellín, Antioquia Colombia; 2 Universidad Nacional de Colombia, Medellín, Antioquia Colombia

**Keywords:** Mesenchymal stem cells, Intrathecal delivery, Safety profile, Systematic review, Meta-analysis

## Abstract

**Background:**

Based on previous in vivo studies and human trials, intrathecal cell delivery is a safe and relevant therapeutic tool for improving patient's quality of life with neurological conditions. We aimed to characterise the safety profile of intrathecally delivered Mesenchymal stem cells (MSCs).

**Methods:**

Ovid MEDLINE, Embase, Scopus, Cochrane Library, KCI-Korean Journal Database, and Web of Science. Databases were searched from their inception until April 13, 2023. Randomised Controlled Trials (RCTs) that compared intrathecal delivery of MSCs to controls in adult populations were included. Adverse events (AEs) were pooled and meta-analysed using DerSimonian-Laird random effects models with a correction factor 0.5 added to studies with zero count cells. Pooled AEs were described using Risk ratio (RR) and 95% confidence intervals (95% CI). Then, a random-effects meta-regress model on study-level summary data was performed to explore the relationship between the occurrence of AEs and covariates thought to modify the overall effect estimate. Finally, publication bias was assessed.

**Results:**

303 records were reviewed, and nine RCTs met the inclusion criteria and were included in the quantitative synthesis (n = 540 patients). MSCs delivered intrathecally, as compared to controls, were associated with an increased probability of AEs of musculoskeletal and connective tissue disorders (categorised by Common Terminology Criteria for Adverse Events—CTCAE version 5.0) (RR: 1.61, 95% CI 1.19–2.19, I^2^ = 0%). The random-effects meta-regress model suggested that fresh MSCs increased the probability of occurrence of AEs compared to cryopreserved MSCs (RR: 1.554; *p*-value = 0.048; 95% CI 1.004–2.404), and the multiple-dose, decreased the probability of AEs by 36% compared to single doses (RR: 0.644; *p*-value = 0.048; 95% CI 0.416–0.996); however, univariate random effects meta-regression models revealed a not significant association between the occurrence of AEs from MSCs intrathecal delivery and each covariate.

**Conclusions:**

Intrathecal delivery of MSCs was associated with a slight increase in AEs associated with musculoskeletal and connective tissue disorders, albeit without serious AEs. We conclude that intrathecal MSCs delivery is safe for patients with neurological conditions. However, further high-quality, large-scale RCTs are needed to confirm these findings.

**Supplementary Information:**

The online version contains supplementary material available at 10.1186/s13287-024-03748-7.

## Introduction

Neurological conditions constitute a wide range of disorders affecting the central and peripheral nervous systems, with reduced cognitive and sensorimotor functioning, representing a prominent global disease burden and a diminished quality of life [1]. With a mortality count of 9·0 million (95% CI 8.8–9.4) and accounting for 16.5% (95% CI 16.1–17.0) of worldwide fatalities, neurological disorders stand as the second most prevalent contributor to mortality following cardiovascular disease [2]. Most neurological disorders are characterised by widespread neuronal death and meagre regenerative potential of the brain [3]. Moreover, the available treatment options are restrained compared with other conditions [4].

MSCs are distinguished by their unique characteristics, such as the capacity for self-renewal and the ability to differentiate into various types of cells [3]. Previous clinical trials have assessed their safety and efficacy as therapies for neurological conditions, including Alzheimer’s disease, dementia, Parkinson’s disease, spinal cord injury, spinal muscular atrophy, and multiple sclerosis [5–10]. MSCs exhibit tropism to injury sites and secrete a diverse range of growth and neurotrophic factors, eliciting immunomodulatory and neuroprotective effects that promote neuronal survival and regeneration [11, 12]. Therefore, MSC therapy could be considered a significant approach for ameliorating neurological dysfunction [13].

MSC therapy can be administered to reach the subarachnoid space through various routes, including intra-arterial, intravenous, intralesional, and intrathecal [7, 14]. Intra-arterial or intravenous routes have been observed to exhibit suboptimal cell delivery into lesion areas, owing to the possible inability of cells to cross the blood–brain barrier (BBB) or potential retention in other organs [10, 15]. Although MSCs have a high potential to cross the BBB, their intrathecal delivery elicits the most significant therapeutic effect, resulting in enhanced cell bioavailability near damaged CNS regions. Furthermore, the apparent absence of significant serious adverse events (SAEs) after intrathecal cell therapy suggests that many cells could be administered without risk [16].

In addition, controlled clinical trials have demonstrated comparable incidences of SAEs between intrathecal injection of MSCs and sham/placebo control groups [15]. Nevertheless, in neurologic MSC therapy studies, the intravenous route remains the most commonly utilised, closely followed by intrathecal injection [17]. This preference may be attributed to ethical considerations arising from the potential risks associated with lumbar puncture and MSCs administration [11]. In this systematic review and meta-analysis, our main objective is to assess the safety profile of intrathecal MSCs administration in adult patients with neurological conditions based on evidence from RCTs that met the inclusion criteria.

## Methods

### Study design

The present study was reported following the Preferred Reporting Items for Systematic Reviews and Meta-Analyses—PRISMA 2020 statement, and the review protocol was registered on Prospero 2023 [CRD42023422142].

### Search strategy and selection criteria

We included only published RCTs limited to human studies available in English. The following electronic databases were searched: Ovid MEDLINE, Embase, Scopus, Cochrane Library, KCI-Korean Journal Database, and Web of Science Databases from their inception until April 13, 2023. Additionally, reference lists were examined by cross-checking the bibliographies of articles and the relevant reviews retrieved. The entire search strategy for all databases is presented in the Supplementary Material.

The inclusion criteria were studies conducted in (a) adults with neurological disorders with (b) intrathecal administration of MSCs from any source or differentiated MSCs (NSC—neuronal stem cell), only or combined with intravascular or intramuscular administration, regardless of cell source; only or combined with standard therapy; (c) control group did not receive MSCs-based therapy; a control group received a placebo, nontreatment, standard treatment or sham procedure; (d) that reported safety outcomes (any AEs associated with MSCs or differentiated MSCs (NSC—neuronal cell) treatment; one AEs reported by more than one study; regardless of the efficacy of MSCs therapy for neurological disorders; and were (e) published RCTs. The inclusion criteria of the studies are summarised in Table 1.

**Table 1:** Inclusion and exclusion criteria

Criteria	Inclusion criteria	Exclusion criteria
Population	Adults with neurological disorders	NA
Intervention	Intrathecal administration of MSCs from any source or differentiated MSCs (NSCs), only or combined with intravascular or intramuscular administration, regardless of cell source; only or combined with standard therapy	MSCs co-administered with other experimental cells such as stem cells from body fluids (blood, urine, serum, tear, saliva, and tissues), ESC, Schwann cells, h-IPS, and olfactory neurons—studies in which MSCs were co-administered with other experimental treatments
Comparison	Control group did not receive MSCs-based therapy; control group received a placebo, nontreatment, standard treatment or sham procedure	Control group that receives any other experimental therapy
Outcome	(1) Any AEs associated with MSC or differentiated MSCs (NSCs) treatment; (2) one AEs reported by more than one study; (3) regardless of the efficacy of MSCs therapy for neurological disorders	Non-AEs reported
Study	Published RCTs	Non-RCTs; studies that exclusively used non-IT routes of administration; MSCs co-administered with other experimental cells or treatments; lacking non-MSCs control group; studies in which hematopoietic stem cells were used; studies in which MSCs were combined with alternative medications or therapies

*MSCs* mesenchymal stem cells, *MNC* mononuclear cells, *NSC* neural stem cells, *ESC* embryonic stem cell, *h-IPS* human-induced pluripotent stem cell, *RCTs* randomised controlled trials, *non-RCTs* non-randomised controlled trials, *AEs* adverse event, *IT* intrathecal, NA not applicable

### Study selection

After deleting duplicated studies using Rayyan software, two independent authors (LEM and JCC) screened the titles and abstracts retrieved through the search strategy and performed the study selection. The full-text versions of all relevant articles were retrieved for the screening process, enabling the assessment of their compliance with the predefined inclusion criteria (LEM and JCC). Disagreements between the two independent reviewers were resolved by discussing or referring to a third reviewer (LLQ) for the final decision. During the data analysis phase, studies lacking information regarding safety outcomes were excluded. Additionally, studies where authors were requested to provide safety-related data via email but failed to do so were excluded from the analysis.

### Data extraction

Two independent authors (LEM and JCC) used a predefined data extraction form to collect the data for each included study. Any disagreements between the two independent reviewers were resolved by a third reviewer (LLQ) for the final decision. Any missing information was requested from the study's corresponding author through e-mail.

### Safety and feasibility

The primary outcome measures included the safety profile evaluated by assessing the occurrences of AEs: an AE was defined as any untoward medical occurrence in a patient, that does not necessarily have a causal relationship with the intrathecal delivery of MSCs treatment. SAEs were considered if they resulted in death or an immediately threatened life, resulted in hospitalisation or longer than anticipated stay in the hospital, or resulted in persistent or significant disability or incapacity. In the context of this study, AEs included any complications associated with the intrathecal delivery of MSCs or differentiated MSCs (NSC—neuronal stem cell) treatment; one AE reported by more than one study, regardless of the efficacy of MSCs therapy for neurological conditions, or serious AEs (SAEs) associated with treatment. The occurrences of AEs were categorised by CTCAE version 5.0 (Common Terminology Criteria for Adverse Events). We used the CONSORT approach for harm reporting to assess the completeness of AEs documentation [18]. Specifically, we examined whether the expected AEs (types of events, frequency, and follow-up) had been monitored and recorded in the methods section. The definitions of safety outcomes are available in Table 2. All the data were extracted at the final follow-up.

**Table 2:** Safety outcomes definition

AEs categorised by CTCAE version 5.0	Definition
General disorders and administration site conditions	(1) Fever (grade 1: 38.0–39.0 °C or 100.4 -102.2 °F) within 24 h. (2) Pain (grade 1, injection site pain)
Musculoskeletal and connective tissue disorders	(1) Back pain (nonspecific, short-duration back pain). (2) Pain in extremities (upper or lower). (3) Arthralgia. (4) Myalgia. (5) Neck pain. (6) Rhabdomyolysis
Nervous system disorders	(1) Dizziness. (2) Headache (nonspecific/positional). (3) Facial nerve disorder (peripheral facial nerve palsy). (4) Muscle weakness
Infections and infestations	(1) Urinary tract infection. (2) Kidney infection (pyelonephritis, acute). (3) Sinusitis. (4) Upper respiratory infection. (5) Viral Infection. (5) Infection distal arm. (6) Scabies infection
Respiratory, thoracic, and mediastinal disorders	(1) Respiratory failure. (2) Cough
Skin and subcutaneous tissue disorders	(1) Rash maculo-papular (facial Rash)
Gastrointestinal disorders	(1) Constipation. (2) Vomiting. (3) Gastrointestinal dysfunction. (4) Nausea
Injury, poisoning and procedural complications	(1) Contusion. (2) Fall. (3) Fracture (leg/hand). (4) Procedural pain. (5) Post-procedural complication. (6) Post-lumbar puncture syndrome (PLPS)
Cardiac disorders	(1) Cardiac arrest
Vascular disorders	(1) Haematoma
Metabolism and nutrition disorders	(1) Hyponatraemia
SAEs	(1) Deaths related to disease progression and other causes, and none were related to study treatment. (2) SAEs related to MS relapses and an upper respiratory infection. (3) All SAEs were deemed to be related to ALS disease progression. (4) Deaths were due to respiratory failure related to disease progression and sudden cardiac arrest

*CTCAE* common terminology criteria for adverse events, *AEs* adverse events, *SAEs* serious adverse events, *ALS* amyotrophic lateral sclerosis, *MS* multiple sclerosis, *°C* degrees Celsius, *°F* degrees Fahrenheit

The data extracted from the RCTs included in this study were related to (a) study characteristics as (first author, publication year, location, number of centres, sample size, and follow-up); (b) patient characteristics (age and sex); (c) characteristics of the MSCs treatment and control treatment: dosage, MSCs sources, origin (autologous or unmatched allogeneic), delivery route, MSCs preparation (fresh or cryopreserved), administration frequency (single or multiple doses) (d) information on the co-therapy.

### Quality assessment and certainty of the evidence

Two independent reviewers (LEM and FBA) assessed the risk of bias for each included study. Discussions resolved disagreements, or a third reviewer (LLQ) was consulted for the final decision. The revised Cochrane risk-of-bias tool for randomised controlled trials was applied for each study included in the systematic review and meta-analysis [19]. We used the Grading of Recommendations Assessment, Development, and Evaluation (GRADE) approach to rate the certainty of evidence for the safety and efficacy outcomes extracted [20].

### Data analysis

Meta-analyses for each pre-specified AE classification were categorised by CTCAE version 5.0 and performed using Stata SE 18—Multivariate meta-analysis software (Stata Corp, Texas, USA). The data were analysed with a random effects model using the DerSimonian-Laird method with the correction of zero-count cells. The pooled dichotomous outcome was described using Relative Risks (RR) with their corresponding 95% confidence intervals (95% CIs). Inconsistency between RCTs was measured using I^2^ and a 10% cut-off for significance [21–23]. Additionally, we used the L’Abbé plot to explore the potential sources of heterogeneity in the meta-analysis. Although we used a random-effects meta-analysis to incorporate heterogeneity among studies, mainly for unexplained heterogeneity, this was not a substitute for a thorough investigation among studies. We considered random-effects meta-regression as an approach to address the clinical and methodological heterogeneity of effect estimates (occurrence of AEs categorised by CTCAE version 5.0) between studies and to explore whether a linear association exists between explanatory variables (as conditions: drug-resistant symptomatic epilepsy, amyotrophic lateral sclerosis (ALS), spinal cord injury (SCI), multiple sclerosis (MS), traumatic brain injury (TBI); MSCs characteristics: cell type, origin, dosage; MSCs preparation: fresh or cryopreserved, xenogeneic or xeno-free medium) and the comparative occurrence of AEs, along with the direction of that association. Finally, we used the GRADE approach to present the safety and efficacy outcomes in a summarised descriptive table.

### Reporting for bias assessment

Publication bias, outcome reporting bias (ORB), and clinical heterogeneity (variability in the participants, interventions, and outcomes) in small studies are also important sources for small-study effects (SSE). Bias analysis was evaluated at the outcome level with a funnel plot and Harbord’s modified test based on regression for small-study effects. A *p-value* < 0.05 suggested evidence for SSE [24].

### Role of funding

The funder provided support through salaries for the authors [LEM, JCC, LLQ, FBA, KH, JAEP]. Nevertheless, it had no additional role in the study design, data collection and analysis, publication decision, or manuscript preparation. The specific roles of these authors are articulated in the “author contributions' section.” Finally, our commercial affiliation did not play a role in this study.

## Results

### Study selection

**Fig. 1:**
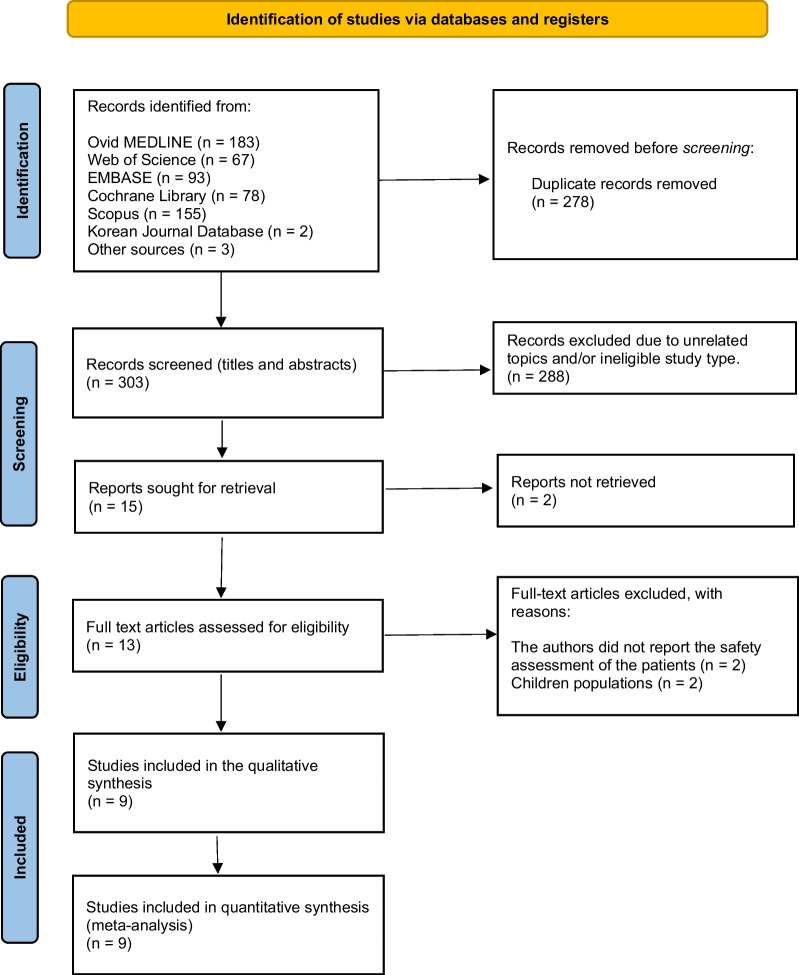
PRISMA flow diagram for the included RCTs

A total of 581 articles were extracted from the literature search. After omitting duplicate studies using an automated tool (Rayyan System, Inc. 2022), 303 articles underwent title and abstract screening. Among these articles, 13 were selected for full-text review. Finally, nine RCTs were included in the study quality assessment and data analysis [7, 9, 10, 12, 17, 25–28]. The literature search and study inclusion process are presented in Fig. 1.

### Study characteristics

Nine RCTs were included (n = 540 patients), and their sample sizes ranged from 10 to 189 patients (60 ± 52.18, mean ± standard deviation); the follow-up periods ranged from one day to 14 months (9.22 ± 3.93, mean ± standard deviation), and the publication dates from 2013 to 2021. The included RCTs were conducted in six different countries: Belarus (two RCTs), the USA (two RCTs), Spain (one RCT), China (two RCTs), Israel (one RCT), and the Republic of Korea (one RCT). Seven studies enrolled patients from a single-centre [7, 10, 12, 17, 25–27], and two were multi-centre [9, 28]. The study characteristics and details about the participants, such as age, sex, intervention and control groups, location, follow-up, clinical conditions, and dosage in the nine RCTs included, are listed in Table 3. Among the included studies, three (33.33%) RCTs used Bone Marrow-derived Mesenchymal Stem Cells (BM-MSCs) [10, 12, 27], two (22.22%) used Umbilical Cord Mesenchymal Stem Cells UC-MSCs [7, 25], and four (44.44%) used Bone Marrow-derived Mesenchymal Stem Cells induced to secrete high levels of Neurotrophic Factors (BMMSCs-NTF) [9, 17, 26, 28]. Concerning the dose, two (22.22%) RCTs administered ≤ 10^6^ cells [17, 26], and seven (77.78%) administered between 10^7^ and 10^8^ cells [7, 9, 10, 12, 25, 27, 28]. Seven (77.78%) RCTs used autologous MSCs [9, 10, 12, 17, 26–28], and two (22.22%) used unmatched allogeneic MSCs [7, 25]. Six (66.67%) RCTs used fresh cells [9, 12, 17, 25–27], and three (33.33%) used cryopreserved MSCs before administration [7, 10, 28]. None of the RCTs included used xenogeneic products, and nine (100%) RCTs cultured the MSCs in a xeno-free medium. Among the 283 patients in the intervention group, 103 received a single dose [7, 9, 17, 26, 27], and 180 received multiple doses [10, 12, 25, 28]. All included RCTs used intrathecal (IT) injection as a treatment delivery route. The most common standard treatments included were Anti-epileptic drugs (AED) such as riluzole. Some important MSCs characteristics, such as the origin (autologous or unmatched allogeneic), MSC preparation (fresh or cryopreserved), xeno-free culture medium, administration frequency (single or multiple doses) and dosage, related to each one of the included studies are detailed in the Supplementary Material (Table S1).

**Table 3:** Study characteristics of intrathecal Mesenchymal Stem Cells-based randomised controlled trials

First author, year	Location country	Clinical population (Sample Size)	Single-centre vs multi-centre	Follow up duration (mos.)	Intervention group	Control group	Mean age (years ± SD)		Sex of patients (female/male)		Analysis	Dosage
							Intervention Group	Control Group	Intervention Group	Control Group		
Hlebokazov Fedor et al., 2021	Belarus	Patients with DRE (n = 67)	Single-centre	12	AEDs supplemented with single IV administration of autologous BM-MSCs, followed by a single IT injection of BMMSC-NTF cells	Conventional AEDs	33·5 ± 10·34	33·4 ± 10·7	20/14	18/15	ITT	Single IV autologous BM-MSCs (1·0 – 1·5 × 10^6^ cells/kg), followed within one week by a single IT injection of BMMSC-NTF (0·1 × 10^6^ cells/kg)
Cudkowicz Merit E., 2022	USA	Patients with ALS (n = 189)	Multi-centre	3	BMMSC-NTF (NurOwn®, manufactured by Dana-Farber Cancer Institute, Boston)	Placebo	48·1 ± 9·71	49·1 ± 8·38	27/68	35/59	ITT	125 × 10^6^ NurOwn® (BMMSC-NTF cells)
Albu Sergiu., 2021	Spain	Patients with complete SCI of traumatic aetiology (n = 10)	Single-centre	12	UC-MSCs first and then placebo infusion	Placebo first and then UC-MSCs infusion	31 ± 6·04	34·4 ± 10·36	2/3	1/4	ITT	10 × 10^6^ UC-MSCs
Song Hua., 2020	China	Patients with acute SCI (n = 36)	Single-centre	12	Control group's treatment + autologous BM-MSCs injection in the subarachnoid space	Treated with decompression + internal fixation + conventional medical treatments	41·2 ± 2·3	41·7 ± 2·1	6/12	8/10	ITT	Autologous 10 × 10^6^ BM-MSCs
Petrou Panayiota., 2020	Israel	Patients with active or worsening progressive MS (n = 64)	Single-centre	14	Autologous BM-MSC and an IV sham injection of normal saline	Sham treatment (normal saline)	Group 1: 49·05 ± 7·2. Group 2: 47·42 ± 10·4	Group 3: 45·89 ± 10·9	Group 1: 6/10. Group 2: 10/6	Group 3: 4/12	ITT	Autologous 1 × 10^6^ BM-MSCs/kg
Berry James D., 2019	USA	Patients with ALS (n = 48)	Multi-centre	6	Autologous BMMSC-NTF	Placebo	50·3 ± 11·90	53·5 ± 9·11	11/25	2/10	ITT	Autologous 125 × 10^6^ BMMSC-NTF cells and 24 IM injections of 48 × 10^6^ BMMSC-NTF at 24 separate sites
Oh, Ki-Wook., 2018	Republic of Korea	Patients with ALS without family history (n = 64)	Single-centre	6	Autologous BM-MSCs at a 26-days interval between applications + Riluzole	Riluzole treatment (100 mg/day)	53·7 ± 7·7	52·5 ± 9·4	14/18	16/11	ITT	Two repeated treatments with IT autologous BM-MSCs (1 × 10^6^ BM-MSC/kg with a 26 days interval)
Hlebokazov Fedor., 2017	Belarus	Patients with DRE (n = 22)	Single-centre	12	AEDs supplemented with single IV administration of autologous BM-MSCs, followed 5–7 days by a single IT injection of BMMSC-NTF	Standard treatment with AEDs	31·5 (20–44) median (range)	32·5 (18–56) median (range)	4/6	5/7	ITT	Autologous 1 × 10^6^ BM-MSCs/kg, followed 5–7 days by a single IT injection of BMMSC-NTF 0.1 × 10^6^ MSC-NTF/kg
Wang Sen., 2013	China	Patients with post-TBI sequelae (n = 40)	Single-centre	6	UC-MSCs group	Control Group	27·50 ± 9·43	28·64 ± 10·13	3/17	5/15	ITT	10 × 10^6^ UC-MSCs

*DRE* drug-resistant epilepsy, *AEDs* anti-epileptic drugs, *ALS* amyotrophic lateral sclerosis, *MS* multiple sclerosis, *SCI* spinal cord injury, *TBI* traumatic brain injury, *BM-MSCs* bone marrow-derived mesenchymal stem cells, *UC-MSCs* umbilical cord mesenchymal stem cells, *BMMSC-NTF* bone marrow-derived mesenchymal stem cells induced to secrete high levels of neurotrophic factor, *MSCs* mesenchymal stem cells, *NTF* neurotrophic factor, *IV* intravenous, IT intrathecal, *PP* per protocol, *ITT* intention to treat, *mos* months

### Risk of bias assessment

**Fig. 2:**
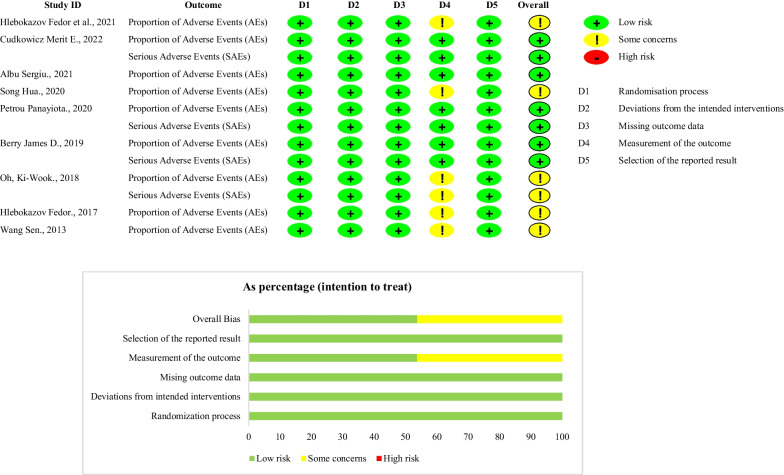
Summary assessment of the risk of bias for the RCTs included using the RoB 2 tool

The risk of bias in the included studies is summarised in Fig. 2. Five studies comprising six safety outcomes had some bias concerns due to outcome measurement (46.2%) [12, 17, 25–27]. For the overall bias, five studies comprising six safety outcomes were defined as having some bias concerns (38.5%) [12, 17, 25–27], and four studies comprising seven outcomes were described as having a low risk of bias (61.5%) [7, 9, 10, 28].

### Publication bias

**Fig. 3:**
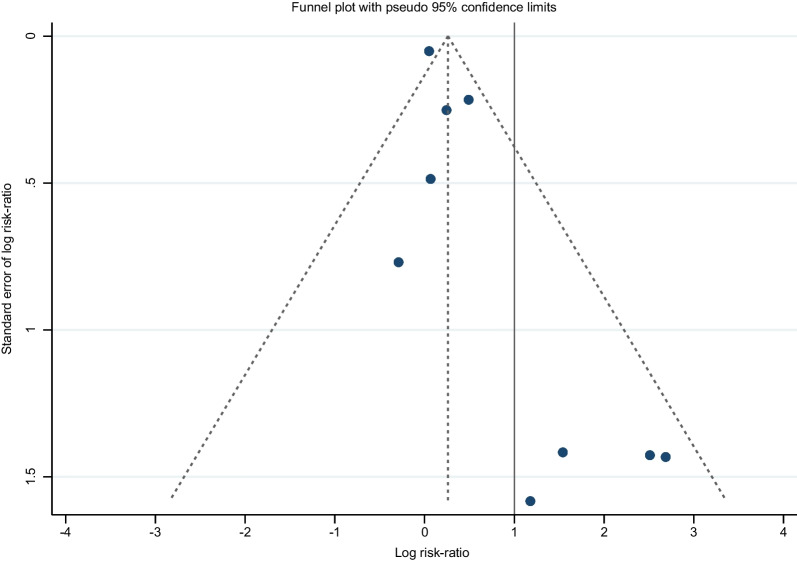
Funnel plot, including all RCTs, shows apparent asymmetry

Publication bias was evaluated with a funnel plot (see Fig. 3), leading to an asymmetrical appearance with a gap on the bottom left-hand side of the graph, which could suggest missing studies. Since this area contains regions of high significance, publication bias is unlikely to be the underlying cause of asymmetry. As a rule, tests for funnel plot asymmetry should only be used when more than ten studies are in the meta-analysis. The power of the test is too low to distinguish chance from real asymmetry, and publication bias cannot be excluded [24, 29]. Therefore, the test results were interpreted in the context of visual inspection of the funnel plot. Despite having only nine RCTs, we performed Harbord’s modified test based on regression for small-study effects, and the test suggested evidence for small-study effects (*p* = 0.021).

### Adverse events (AEs) and serious adverse events (SAEs)

**Fig. 4:**
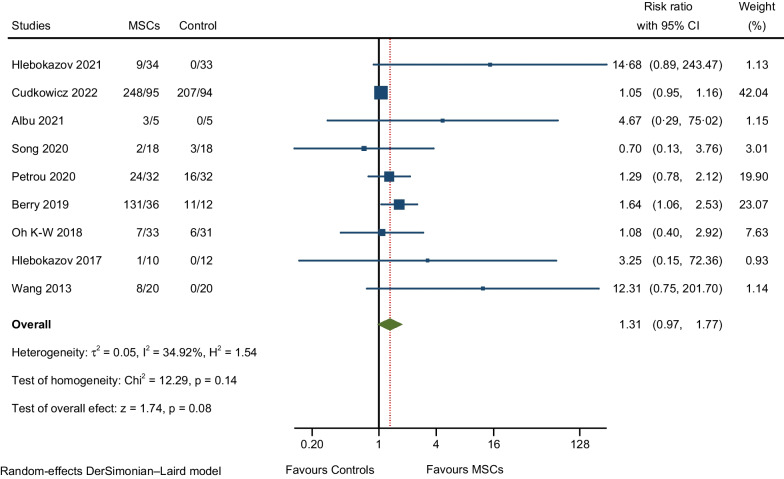
Meta-analysis of overall AEs

**Fig. 5:**
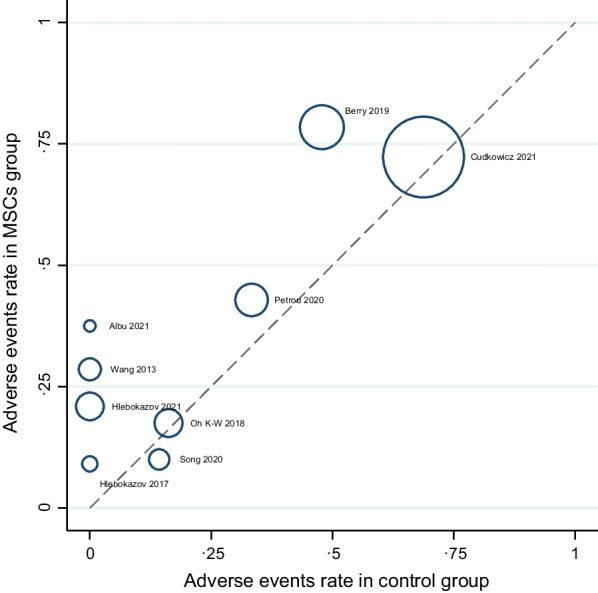
L’Abbé plot for overall AEs in the MSCs and control groups

**Fig. 6:**
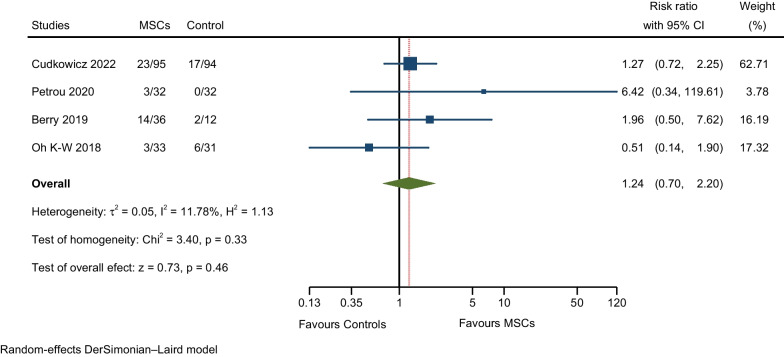
Meta-analysis of SAEs

A description and frequency of AEs and SAEs are provided in Tables 2, 4, and 5. The meta-analysis of overall AEs for all RCTs included in this study showed a not significantly higher rate of AEs in the MSCs group compared to the control group (RR: 1.31; 95% CI 0.97–1.77; I^2^ = 34.92%; Fig. 4) [7, 9, 10, 12, 17, 25–28]. We assessed heterogeneity by comparing the rate of overall AEs between the MSCs and control groups (Fig. 5); each circle in the L’Abbé plot represents an individual RCT, and larger circles represent RCTs with more AEs; the dotted diagonal line indicates that the rate of overall AEs is equal in the two groups within RCTs. The overall AEs rates exhibited significant variability in the MSCs group (0.23–57.27%) and the control group (0–85.19%). The highest rate of overall AEs in the MSCs group was reported in the RCT by Cudkowicz et al. (57.27%). On the other hand, the lowest rate of overall AEs in the MSCs group was observed in the trial by Hlebokazov et al. (0.231%). Four RCTs reported SAEs; Cudkowicz et al. reported deaths related to disease progression and other causes unrelated to the study intervention; Petrou et al. adjudged SAEs to MS relapses and an upper respiratory infection; Berry et al. reported that all SAEs were related to ALS disease progression; and Oh K-W et al., regarded deaths as due to respiratory failure related to disease progression and sudden cardiac arrest. The meta-analysis of the pooled effect estimates showed non-significant differences in the occurrence of SAEs in the MSCs group compared to the control group (RR: 1.24; 95% CI 0.70–2.20; I^2^ = 11.78%; Fig. 6) [9, 10, 12, 28]. Table 6 provides a comprehensive summary of the safety outcomes and the quality of the safety reporting findings.

**Table 4:** Number of incident AEs by author and year

AEs categorised by CTCAE version 5.0	Statements of safety and AEs reported	Frequency MSCs Group		Frequency control group	
		AEs	No. of patients (%)	AEs	No. of patients (%)
General disorders and administration site conditions		27 (6.24%)	135 (47.70%)	3 (1.23%)	108 (42.02%)
Hlebokazov Fedor et al., 2021	Fever (within 24 h after MSCs injection)*	3	34	0	33
Petrou P et al., 2020	Fever (within 24 h after MSCs injection)	1	32	2	32
Berry JD et al., 2019 (a)	Fever (within 24 h after MSCs injection)	12	36	0	12
Berry JD et al., 2019 (b)	Pain (injection site pain)	10	36	1	12
Oh Ki-Wook et al., 2018	Fever (within 24 h after MSCs injection)	1	33	0	31
Musculoskeletal and connective tissue disorders		139 (32.10%)	235 (83.04%)	48 (19.75%)	207 (80.54%)
Hlebokazov Fedor et al., 2021	Pain in extremities (upper or lower)*	3	34	0	33
Cudkowicz ME et al., 2022 (a)	Pain in extremities (upper or lower)	31	95	19	94
Cudkowicz ME et al., 2022 (b)	Back pain (nonspecific, short-duration back pain)	42	95	24	94
Albu Sergiu et al., 2021	Back pain (nonspecific, short-duration back pain)	1	5	0	5
Petrou P et al., 2020	Back pain (nonspecific, short-duration back pain)	2	32	3	32
Berry JD et al., 2019 (a)	Arthralgia	12	36	0	12
Berry JD et al., 2019 (b)	Neck pain	7	36	0	12
Berry JD et al., 2019 (c)	Pain in extremities (upper or lower)	8	36	0	12
Berry JD et al., 2019 (d)	Back pain (nonspecific, short-duration back pain)	26	36	1	12
Berry JD et al., 2019 (e)	Myalgia	6	36	0	12
Oh Ki-Wook et al., 2018 (a)	Rhabdomyolysis	0	33	1	31
Oh Ki-Wook et al., 2018 (b)	Pain in extremities (upper or lower)	1	33	0	31
Nervous system disorders		99 (22.86%)	283 (100%)	59 (24.28%)	257 (100%)
Hlebokazov Fedor et al., 2021	Headache (nonspecific/positional)*	3	34	0	33
Cudkowicz ME et al., 2022 (a)	Headache (nonspecific/positional)	31	95	30	94
Cudkowicz ME et al., 2022 (b)	Muscular weakness	11	95	12	94
Albu Sergiu et al., 2021	Headache (nonspecific/positional)	1	5	0	5
Song Hua et al., 2020	Headache (nonspecific/positional)	1	18	1	18
Petrou P et al., 2020 (a)	Headache (nonspecific/positional)	9	32	8	32
Petrou P et al., 2020 (b)	Dizziness	2	32	0	32
Petrou P et al., 2020 (c)	Facial nerve disorder (peripheral facial nerve palsy)	1	32	0	32
Berry JD et al., 2019	Headache (nonspecific/positional)	29	36	8	12
Oh Ki-Wook et al., 2018	Headache (nonspecific/positional)	2	33	0	31
Hlebokazov Fedor et al., 2017	Headache (nonspecific/positional)	1	10	0	12
Wang Sen et al., 2013 (a)	Headache (nonspecific/positional)	4	20	0	20
Wang Sen et al., 2013 (b)	Dizziness	4	20	0	20
Infections and infestations		6 (1.39%)	83 (29.33%)	3 (1.23%)	81 (31.52%)
Song Hua et al., 2020	Urinary tract infection	0	18	1	18
Petrou P et al., 2020 (a)	Viral Infection	0	32	1	32
Petrou P et al., 2020 (b)	Upper respiratory infection	1	32	0	32
Petrou P et al., 2020 (c)	Sinusitis	2	32	0	32
Petrou P et al., 2020 (d)	Scabies infection	1	32	0	32
Petrou P et al., 2020 (e)	Infection distal arm	0	32	1	32
Petrou P et al., 2020 (f)	Urinary tract infection	1	32	0	32
Oh Ki-Wook et al., 2018	Kidney infection	1	33	0	31
Respiratory, thoracic, and mediastinal disorders		7 (1.62%)	69 (24.38%)	2 (0.82%)	43 (16.73%)
Berry JD et al., 2019	Cough	6	36	0	12
Oh Ki-Wook et al., 2018	Respiratory failure	1	33	2	31
Skin and subcutaneous tissue disorders		1 (0.23%)	32 (11.31%)	0 (0%)	32 (12.45%)
Petrou P et al., 2020	Rash maculo-papular (facial Rash)	1	32	0	32
Gastrointestinal disorders		33 (7.62%)	154 (54.42%)	20 (8.23%)	129 (50.19%)
Cudkowicz ME et al., 2022	Nausea	16	95	18	94
Albu Sergiu et al., 2021	Vomiting	1	5	0	5
Song Hua et al., 2020	Gastrointestinal dysfunction	1	18	1	18
Berry JD et al., 2019 (a)	Constipation	9	36	1	12
Berry JD et al., 2019 (b)	Nausea	6	36	0	12
Injury, poisoning and procedural complications		119 (27.48%)	160 (56.54%)	107 (44.03%)	157 (61.09%)
Cudkowicz ME et al., 2022 (a)	Procedural pain	50	95	34	94
Cudkowicz ME et al., 2022 (b)	Post-procedural complication	16	95	7	94
Cudkowicz ME et al., 2022 (c)	Post-lumbar puncture syndrome	22	95	29	94
Cudkowicz ME et al., 2022 (d)	Fall	29	95	34	94
Petrou P et al., 2020 (a)	Fall	1	32	1	32
Petrou P et al., 2020 (b)	Fracture (leg/hand)	1	32	0	32
Oh Ki-Wook et al., 2018 (a)	Fall	0	33	1	31
Oh Ki-Wook et al., 2018 (b)	Contusion	0	33	1	31
Cardiac disorders		0 (0%)	33 (11.66%)	1 (0.41%)	31 (12.06%)
Oh Ki-Wook et al., 2018	Cardiac arrest	0	33	1	31
Vascular disorders		1 (0.23%)	32 (11.31%)	0 (0%)	32 (12.45%)
Petrou P et al., 2020	Haematoma	1	32	0	32
Metabolism and nutrition disorders		1 (0.23%)	33 (11.66%)	0 (0%)	31 (12.06%)
Oh Ki-Wook et al., 2018	Hyponatraemia	1	33	0	31
SAEs, total		43 (9.93%)	196 (69.26%)	25 (10.29%)	169 (65.76%)
Cudkowicz ME et al., 2022	None of the SAEs related to disease progression and other causes were considered related to study treatment (ten deaths not related to study treatment in the MSCs group and six in the Control group)	23	95	17	94
Petrou P et al., 2020	SAEs related to MS relapses and an upper respiratory infection	3	32	0	32
Berry JD et al., 2019	All SAEs were deemed to be related to ALS disease progression	14	36	2	12
Oh Ki-Wook et al., 2018	Four deaths occurred during the trial; three (one MSC-treated participant and two control participants) were caused by respiratory failure related to disease progression, and one in the control group was caused by sudden cardiac arrest	3	33	6	31

*CTCAE* common terminology criteria for adverse events, *AEs* adverse events, *SAEs* serious adverse events, *MSCs* mesenchymal stem cells, *RCTs* randomised controlled trials, *ALS* amyotrophic lateral sclerosis, *MS* multiple sclerosis

*Information obtained via correspondence with the author

**Table 5:** AEs and SAEs for all participants from the first intrathecal MSCs injection to the last follow-up

AEs categorised by CTCAE version 5.0	AEs (from + 0 to the last follow-up period)			
	Frequency treatment group		Frequency control group	
	Total no. %)	No. of patients (%)	Total no. (%)	No. of patients (%)
General disorders and administration site conditions	27 (6.24%)	135 (47.70%)	3 (1.23%)	108 (42.02%)
Fever (within 24 h after MSCs injection)	17	135	2	108
Pain (injection site pain)	10	36	1	12
Musculoskeletal and connective tissue disorders	139 (32.10%)	235 (83.04%)	48 (19.75%)	207 (80.54%)
Back pain (nonspecific, short-duration back pain)	71	168	28	143
Pain in extremities (upper or lower)	43	198	19	170
Arthralgia	12	36	0	12
Myalgia	6	36	0	12
Neck pain	7	36	0	12
Rhabdomyolysis	0	33	1	31
Nervous system disorders	99 (22.86%)	283 (100%)	59 (24.28%)	257 (100%)
Dizziness	6	52	0	52
Headache (nonspecific/positional)	81	283	47	257
Facial nerve disorder (peripheral facial nerve palsy)	1	32	0	32
Muscle weakness	11	95	12	94
Infections and infestations	6 (1.39%)	83 (29.33%)	3 (1.23%)	81 (31.52%)
Urinary tract infection	1	50	1	50
Kidney infection (pyelonephritis, acute)	1	33	0	31
Sinusitis	2	32	0	32
Upper respiratory infection	1	32	0	32
Viral Infection	0	32	1	32
Infection distal arm	0	32	1	32
Scabies infection	1	32	0	32
Respiratory, thoracic, and mediastinal disorders	7 (1.62%)	69 (24.38%)	2 (0.82%)	43 (16.73%)
Respiratory failure	1	33	2	31
Cough	6	36	0	12
Skin and subcutaneous tissue disorders	1 (0.23%)	32 (11.31%)	0 (0%)	32 (12.45%)
Rash maculo-papular (facial Rash)	1	32	0	32
Gastrointestinal disorders	33 (7.62%)	154 (54.42%)	20 (8.23%)	129 (50.19%)
Constipation	9	36	1	12
Vomiting	1	5	0	5
Gastrointestinal dysfunction	1	18	1	18
Nausea	22	131	18	106
Injury, poisoning and procedural complications	119 (27.48%)	160 (56.54%)	107 (44.03%)	157 (61.09%)
Contusion	0	33	1	31
Fall	30	160	36	157
Fracture (leg/hand)	1	32	0	32
Procedural pain	50	95	34	94
Post-procedural complication	16	95	7	94
Post-lumbar puncture syndrome (PLPS)	22	95	29	94
Cardiac disorders	0 (0%)	33 (11.66%)	1 (0.41%)	31 (12.06%)
Cardiac arrest	0	33	1	31
Vascular disorders	1 (0.23%)	32 (11.31%)	0 (0%)	32 (12.45%)
Haematoma	1	32	0	32
Metabolism and nutrition disorders	1 (0.23%)	33 (11.66%)	0 (0%)	31 (12.06%)
Hyponatraemia	1	33	0	31
SAEs, total	43 (9.93%)	196 (69.26%)	25 (10.29%)	169 (65.76%)
AEs, total	433 (100%)	283 (100%)	243 (100%)	257 (100%)

*CTCAE* common terminology criteria for adverse events, *AEs* adverse events, *SAEs* serious adverse events

**Table 6:** Safety outcomes and quality of safety reporting findings

Safety outcomes	# of RCTs*	Findings (RR; 95% CI)	Heterogeneity I^2^
General disorders and administration site conditions	4/9	2.07 (0.76–64.94)	0.00%
Musculoskeletal and connective tissue disorders	6/9	1.61 (1.19–2.19)	0.00%
Nervous system disorders	9/9	1.16 (0.87–1.54)	0.00%
Infections and infestations	3/9	1.40 (0.46–4.25)	0.00%
Respiratory, thoracic, and mediastinal disorders	2/9	1.19 (0.16–9.08)	20.14%
Skin and subcutaneous tissue disorders	1/9	NA	NA
Gastrointestinal disorders	4/9	1.07 (0.63–1.83)	0.00%
Injury, poisoning and procedural complications	3/9	1.07 (0.85–1.34)	0.00%
Cardiac disorders	1/9	NA	NA
Vascular disorders	1/9	NA	NA
Metabolism and nutrition disorders	1/9	NA	NA
SAEs, total	4/9	1.24 (0.70–2.20)	11.78%
AEs, total	9/9	1.31 (0.97–1.77)	34.92%
Quality of safety reporting	# of RCTs*	Findings (%)	NA
A priori plan to monitor adverse events	9/9	100%	NA

*Trials that reported AEs

*RCTs* randomised controlled trials, *AEs* adverse events, *SAEs* serious adverse events, *NA* not applicable

### System organ classes (SOC) related AEs

#### AEs related to general disorders and administration site conditions

**Fig. 7:**
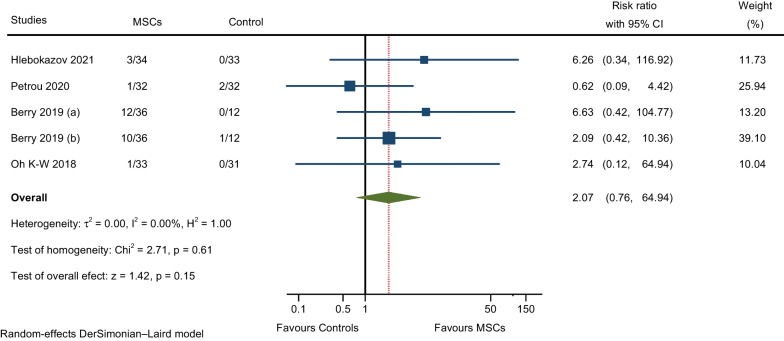
Meta-analysis of AEs related to general disorders and administration site conditions

Meta-analysis of four RCTs that reported fever (within 24 h after MSCs injection) and pain (injection site pain) showed a not significant, slightly higher rate of AEs with the MSCs group when compared to the control group (RR: 2.07; 95% CI 0.76–64.94; I^2^ = 0%; Fig. 7) [9, 10, 12, 17].

#### AEs related to musculoskeletal and connective tissue disorders

**Fig. 8:**
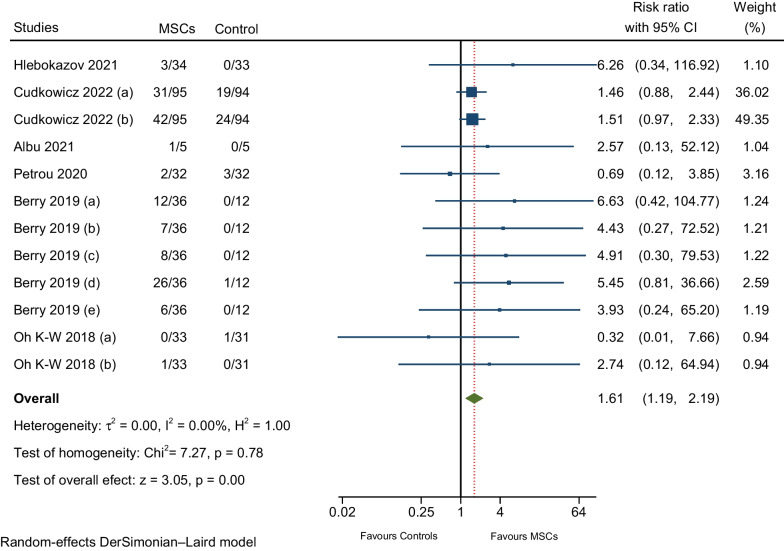
Meta-analysis of AEs related to musculoskeletal and connective tissue disorders

Meta-analysis of six RCTs reported the following AEs: (i) pain in extremities—upper or lower (four RCTs), (ii) back pain—nonspecific, short duration back pain (four 4 RCTs), (iii) arthralgia (one RCT), (iv) myalgia (one RCT), (v) rhabdomyolysis (one RCT), and (vi) neck pain (one RCT). The pooled estimate of the individual effect sizes of the primary RCTs revealed a significant rate of AEs was slightly higher with MScs compared to the control group (RR: 1.61; 95% CI 1.19–2.19; I^2^ = 0%; Fig. 8) [7, 9, 10, 12, 17, 28].

#### AEs related to nervous system disorders

**Fig. 9:**
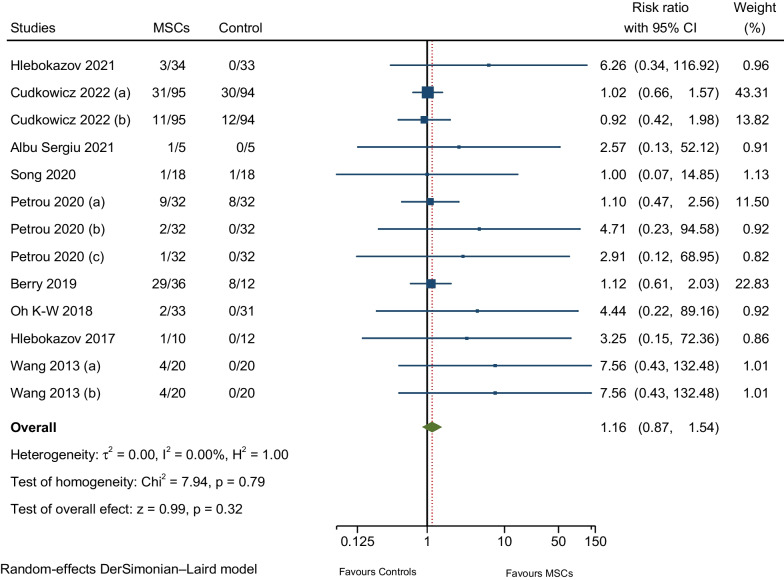
Meta-analysis of AEs related to nervous system disorders

Nine RCTs reported headache—nonspecific/positional; one RCT reported muscular weakness; two RCTs reported dizziness; and another RCT informed facial nerve disorder—peripheral facial nerve palsy. Meta-analysis of pooled estimates for AEs related to nervous system disorders revealed a slightly higher rate in the MSCs group compared to the placebo group, but the difference was not significant (RR: 1.16; 95% CI 0.87–1.54; I^2^ = 0%; Fig. 9) [7, 9, 10, 12, 17, 25–28].

#### AEs related to infections and infestations

**Fig. 10:**
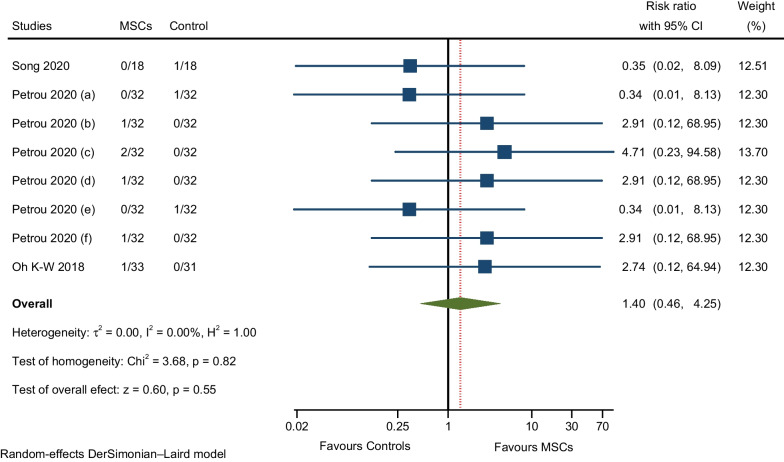
Meta-analysis of AEs related to infections and infestations

Meta-analysis of three RCTs reported the occurrence of urinary tract infection (two RCTs), viral infection (one RCT), upper respiratory infection (one RCT), sinusitis (one RCT), scabies infection (one RCT), infection in the distal arm (one RCT), and kidney infection (one RCT). The pooled estimate of effect size revealed a not significant, slightly higher rate of AEs in the MSCs group compared to the control group (RR: 1.40; 95% CI 0.46–4.25; I^2^ = 0%; Fig. 10) [10, 12, 27].

#### AEs related to respiratory, thoracic, and mediastinal disorders

**Fig. 11:**
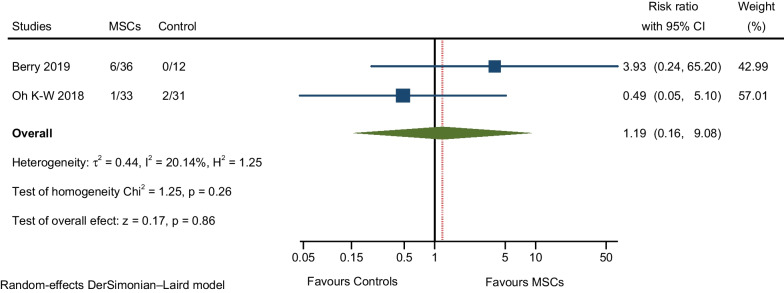
Meta-analysis of AEs related to respiratory, thoracic, and mediastinal disorders

Two RCTs reported respiratory failure and cough (both related to disease progression). A pooled analysis detected a higher rate of AEs in the MSCs group compared to the control group, but the difference between the two groups was not significant (RR: 1.19; 95% CI 0.16–9.08; I^2^ = 20.14%; Fig. 11) [9, 12].

#### AEs related to gastrointestinal disorders

**Fig. 12:**
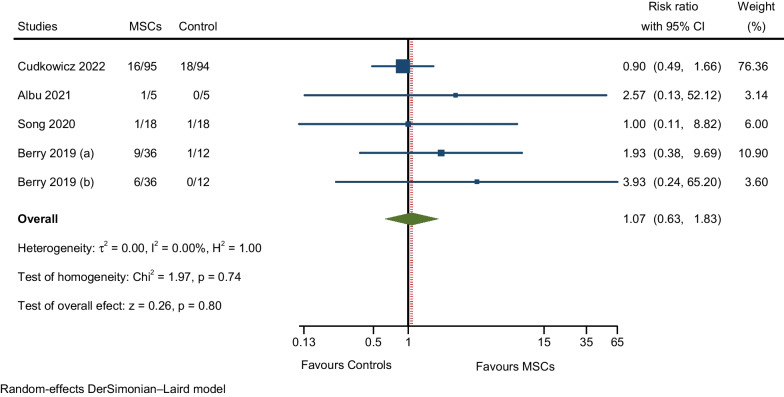
Meta-analysis of AEs related to gastrointestinal disorders

Four RCTs reported AEs such as nausea, vomiting, gastrointestinal dysfunction, and constipation. The results of the meta-analysis showed that the rate of AEs was slightly higher with MSCs compared to the control group, but the difference was not significant (RR: 1.07; 95% CI 0.63–1.83; I^2^ = 0%; Fig. 12) [7, 9, 27, 28].

#### AEs related to injury, poisoning, and procedural complications

**Fig. 13:**
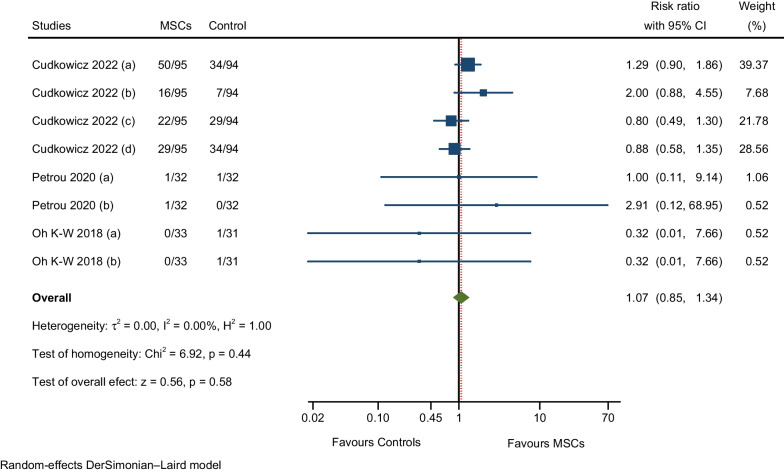
Meta-analysis of AEs related to injury, poisoning, and procedural complications

The pooled estimate of the effect size of the three RCTs revealed a not significantly higher rate of AEs in the MSCs group than in the control group (RR: 1.07; 95% CI 0.85–1.34; I^2^ = 0%; Fig. 13). The AEs reported in three RCTs were procedural pain, post-procedural complications, post-lumbar puncture syndrome, falls, fractures (leg/hand), and contusions [10, 12, 28].

#### AEs related to cardiac disorders

One RCT reported cardiac arrest (not considered to be treatment-related) [12].

#### AEs related to vascular disorders

One RCT reported a Haematoma (unrelated to the procedure or treatment) [10].

#### AEs related to metabolism and nutrition disorders

One RCT reported hyponatraemia (not considered treatment-related) [12].

### Model of probability *p* of AEs occurrence with intrathecal MSCs therapy

We performed a comprehensive random effects meta-regression model instead of a subgroup analysis because the meta-regression method efficiently allows the evaluation of one or more covariates simultaneously and explores the sources of clinical or methodological heterogeneity of treatment effects across RCTs. The models obtained are described in Table 7. The pooled estimate of AEs associated with musculoskeletal and connective tissue disorders demonstrated a significantly higher rate in the MSCs group compared to the control group. (RR: 1.61; 95% CI 1.19–2.19; Fig. 7), while a Cochran’s Q statistics test (*p* = 0.78) and an I^2^ = 0% suggested homogeneity between studies; however, we were able to show differences in the rate of AEs occurrence (treatment effect) based on the MSCs preparation (fresh or cryopreserved) and administration frequency (single or multiple doses) when we conducted the meta-regression. Upon meta-regression, we demonstrated a significant positive association between the probability *p* of AE occurrence with intrathecal MSCs therapy when fresh MSCs were used compared to cryopreserved cells. (RR: 1.554; 95% CI 1.004–2.404; *p* = 0.048*; R-squared = 100% ‘best fit’ line; Model 2). On the other hand, in Model 3, the meta-regression suggested that the probability *p* of AE occurrence resulting from RCTs that delivered multiple doses of MSCs decreased (36%) compared to the RCTs that offered a single dose of cells (RR: 0.644; 95% CI 0.416–0.996; *p* = 0.048*; R-squared = 100% ‘best fit’ line; Model 3). However, the joint test for all three covariates yielded a *p*-value (Model 2) of 0.106 and a p-value (Model 3) of 0.106, indicating that no evidence existed for an association of at least one of the three covariates with the size of the treatment effect (AEs occurrence). Additionally, we performed univariate random effects meta-regression models of the probability *p* of AE occurrence with intrathecal MSCs therapy to control the higher likelihood of false-positive findings when performing meta-regression with multiple covariates. Nevertheless, the results of the meta-regress did not show evidence for an association between the rate of AE occurrence and any covariate included in the analysis (univariate models can be found in Supplementary Material Table S2).

**Table 7:** Meta-regression summary

Probability of AEs occurrence with IT MSCs therapy	Model 1 R-squared (%) = 100 I^2^ (%) = 0.00 Prob > Chi^2^ = 0.106	Model 2 R-squared (%) = 100 I^2^ (%) = 0.00 Prob > Chi^2^ = 0.106	Model 3 R-squared (%) = 100 I^2^ (%) = 0.00 Prob > Chi^2^ = 0.106	Model 4 R-squared (%) = 6.78 I^2^ (%) = 10.06 Prob > Chi^2^ = 0.359
*Cell Type, BM-MSCs (base)*				
BMMSC-NTF (RR)	9.950	1.528	0.983	1.059
*p*-Value (95% CI)	0.080 (0.760–130.184)	0.426 (0.538–4.336)	0.972 (0.377–2.563)	0.871 (0.529–2.122)
UC-MSCs (RR)	63.115	9.690	6.236	5.910
*p*-Value (95% CI)	0.084 (0.575–6927.39)	0.163 (0.399–235.501)	0.256 (0.264–147.097)	0.096 (0.731–47.798)
*MSCs preparation, Cryopreserved (base)*				
Fresh (RR)	10.120	1.554	…	1.159
*p*-Value (95% CI)	0.098 (0.651–157.395)	0.048* (1.004–2.404)	…	0.782 (0.407–3.30)
*Administration Frequency, Single dose (base)*				
Multiple dose (RR)	6.514	…	0.644	0.804
*p*-Value (95% CI)	0.186 (0.406–104.475)	…	0.048* (0.416–0.996)	0.711 (0.255–2.542)
*Clinical Population, SCI (base)*				
MS (RR)	2.650	2.650	2.650	…
*p*-Value (95% CI)	0.246 (0.511–13.752)	0.246 (0.511–13.752)	0.246 (0.511–13.752)	…
ALS (RR)	0.219	1.429	2.220	…
*p*-Value (95% CI)	0.162 (0.026–1.837)	0.695 (0.240–8.514)	0.395 (0.354–13.943)	…
TBI (RR)	0.040	1.698	4.099	…
*p*-Value (95% CI)	0.351 (0.000–34.796)	0.794 (0.032–89.532)	0.486 (0.078–216.136)	…
DRE (RR)	…	6.514	10.120	…
*p*-Value (95% CI)	…	0.186 (0.406–104.475)	0.098 (0.651–157.395)	…
Constant (RR)	0.074	0.482	0.748	1.324
*p*-Value (95% CI)	0.178 (0.02–3.271)	0.362 (0.100–2.317)	0.706 (0.165–3.383)	0.704 (0.311–5.633)

*AEs* adverse events, *MSCs* mesenchymal stem cells, *MS* multiple sclerosis, *ALS* amyotrophic lateral sclerosis, *TBI* traumatic brain injury, *SCI* spinal cord injury, *IT* intrathecal, DRE drug-resistant epilepsy, *RR* risk-ratio

*Statistical significance: *p* < 0·05

### Certainty of the evidence (GRADE assessment)

We used the GRADE approach to rate the quality of evidence for each critical outcome of our study's safety and efficacy profile. We developed and presented summary findings for all comparisons between MSC therapy and control interventions in the included RCTs.

The quality of evidence was downgraded for risk of bias, inconsistency, and imprecision of the results. The certainty of evidence of the proportion of patients with AEs outcomes in the MSCs group and control group was moderate (outcome measures were reported in 100% of RCTs); the certainty of the proportion of patients with SAEs in the MSCs group and control group was moderate (outcome measures were reported in four (44.4%) RCTs); the certainty of the proportion of patients with AEs outcomes at general disorders and administration site conditions (AEs categorised by CTCAE version 5.0) in the MSCs group and control group was moderate (outcome measures were reported in four (44.4%) RCTs); the certainty of the proportion of patients with AEs outcomes at musculoskeletal and connective tissue disorders in the MSCs group and control group was high (outcome measures were reported in four (66.67%) RCTs); the certainty of the proportion of patients with AEs outcomes at nervous system disorders in the MSCs group and control group was moderate (outcome measures were reported in 100% RCTs); the certainty of the proportion of patients with AEs outcomes at infections and infestations in the MSCs group and control group was moderate (outcome measures were reported in three (33.33%) RCTs); the certainty of the proportion of patients with AEs outcomes at respiratory, thoracic and mediastinal disorders in the MSCs group and control group was moderate (outcome measures were reported in two (22.22%) RCTs); the certainty of the proportion of patients with AEs outcomes at gastrointestinal disorders in the MSCs group and control group was moderate (outcome measures were reported in four (44.44%) RCTs); the certainty of the proportion of patients with AEs outcomes at injury, poisoning and procedural complications in the MSCs group and control group was moderate (outcome measures were reported in three (33.33%) RCTs); For all safety outcomes, downgrading of certainty of evidence due to imprecision were (serious, 1 level); except for AEs related to musculoskeletal and connective tissue disorders where was high. The certainty of evidence for the primary efficacy outcomes summarised can be seen in Supplementary Material Table S3.

### Current status and ongoing clinical trials that included a control group

We also searched ongoing trials in the following websites and online databases of clinical research studies (last searched July 28, 2023): the US National Institutes of Health Ongoing Trials Register ClinicalTrials.gov (www.clinicaltrials.gov); the World Health Organization International Clinical Trials Registry Platform (apps.who.int/trialsearch) and, The European Union Clinical Trials Register (www.clinicaltrialsregister.eu). The unpublished findings are shown in Supplementary Material Table S4, complementing the current information.

## Discussion

To the best of our knowledge, this study is the first systematic review and meta-analysis that summarises the safety profile of the intrathecal delivery of MSCs and supports the findings of trials designed to evaluate the safety and efficacy of single or multiple doses of intrathecal MSCs treatment in which the authors suggest that this route of cell administration is a potentially safe treatment for patients with neurological conditions [7, 9, 10, 12, 17, 25–28]. However, the results of the meta-analysis showed that the pooled estimate of the individual effect sizes of RCTs revealed that a significant rate of AEs related to musculoskeletal and connective tissue disorders was slightly higher with MSCs when compared to the control group; our univariate random effects meta-regression models were not able to demonstrate associations between the rate of AEs occurrence and the covariates included in the analysis. However, it should also be emphasised that these analyses (meta-regression) are exploratory, and the magnitude of the effect and its 95% confidence interval are more relevant than whether a p-value is less than or greater than 0.05. In addition to statistical significance, clinical relevance is essential, especially in systematic reviews and meta-analyses, where only associations, not causality, can be demonstrated [30].

Several studies reported the occurrence of non-SAE, transient AEs concerning the local response to the therapy and the lumbar puncture procedure; these AEs were categorised by CTCAE version 5.0 within general disorders and administration site conditions. Our findings demonstrated that the occurrence of AEs, such as fever and pain at the injection site, was not significant (RR: 2.07; 95% CI 0.76–64.94) compared to the control group that received placebo or conventional therapy. It is important to note that the symptoms related to the occurrence of AEs categorised by CTCAE, such as musculoskeletal and connective tissue disorders, which had a significantly higher rate of AEs (back pain -nonspecific, short-duration back pain; pain in extremities; arthralgia; myalgia; neck pain) give rise to minimal risk to patients undergoing MSCs therapy if they are monitored closely in the early days after intrathecal application.

Our systematic review and meta-analysis were performed using a comprehensive methodology explicitly tailored to studying the safety profile of MSC therapy. We used a wide range of electronic databases to reduce the potential for publication bias and provide a general overview of the literature. While other systematic reviews have also supported the safety of MSC therapy delivered through different routes, our study is notable because it utilises statistical techniques to pool safety data related to the intrathecal delivery of MSCs on a common endpoint for nine RCTs included.

The present study has several limitations that must be acknowledged. First, Harbord’s modified test based on regression for small-study effects suggested evidence supporting such effects (*p* = 0.021), confirmed following a visual inspection of the funnel plot (see Fig. 3). However, smaller studies may also be biased because they are targeted at high-risk groups (as in our case, patients with neurological conditions), where the MSCs therapy is likely beneficial, resulting in a trend for smaller studies to show higher treatment effects. Second, we included two RCTs [7, 10] that utilised a crossover design. However, this design can be problematic due to the potential for compromised blinding from the treatment effects and for the probability of the patients randomised in the first step not returning to the baseline conditions before the second crossover (carryover effects). In the case of these two RCTs, these concerns were mitigated by authors using a control group such as placebo infusion [7] or placebo sham treatment [10] and using ample time–space (six months) between the first and second cycle of MSC treatment [7, 10].

## Conclusions

In summary, the administration of MSCs via intrathecal delivery resulted in a minor increase in AEs linked to musculoskeletal and connective tissue disorders. However, no SAEs were observed, confirming findings from prior clinical trials showing that the intrathecal injection of MSCs may be a safe delivery route for patients with neurological conditions. Limited by the evidence for small-study effects and the crossover design of trials that can be problematic, large sample sizes and well-designed multi-centre RCTs with long follow-up periods are required in future analyses.

### Supplementary Information

Supplementary Material. Supplementary S1–S4. Data related to this study. Supplementary S5. PRISMA checklist.

## Data Availability

The data collected for this systematic review and meta-analysis are secondary and are available in the original published trials. Pooled data through meta-analysis will be available within the manuscript or Supplementary Material. Additional information can be accessed through the corresponding author upon request.
