# Supplementary material for: The safety profile of mesenchymal stem cell therapy administered through intrathecal injections for treating neurological disorders: a systematic review and meta-analysis of randomised controlled trials
Source: Stem Cell Res Ther. 2024 May 20;15:146. doi: 10.1186/s13287-024-03748-7 (PMC11103979; doi:10.1186/s13287-024-03748-7)
Supplement: Supplementary file 1 — Supplementary Material. Supplementary S1–S4. Data related to this study. Supplementary S5. PRISMA checklist. [file 13287_2024_3748_MOESM1_ESM.docx]

**The safety profile of Mesenchymal Stem Cell therapy administered through intrathecal injections for treating neurological disorders: a systematic review and meta-analysis of randomised controlled trials.**

SUPPLEMENTARY MATERIAL

**TABLE OF CONTENTS**

**Ovid MEDLINE3**

Research Strategy3

**Embase3**

Research Strategy3

**Scopus4**

Research Strategy4

**Cochrane Library4**

Research Strategy4

**KCI – Korean Journal Database 5**

Research Strategy5

**Web of Science5**

Research Strategy5

**Supplementary Table S1. MSCs Characteristics 6**

**Supplementary Table S2. Univariate random effects meta-regression models**.**7**

**Supplementary Table S3. Grade approach to rate the certainty from a systematic review and meta-analysis.8**

**Supplementary Table S4. Summary of active intrathecal MSCs-based clinical trials that included a control group.**…**11**

**Ovid MEDLINE**

Research Strategy: Ovid MEDLINE(R) / PubMed(R) 1946 to Present

("safe" or "safety" or "side event$1" or "side effect$1" or ("side" and "event$1") or ("side" and "effect$1") or "adverse event$1" or "adverse effect$1" or ("adverse" and "event$1") or ("adverse" and "effect$1") or ((adverse or undesirable or harm* or serious or toxic) adj3 (effect* or reaction* or event* or outcome*))).tw,ti,ab,mp. 3451673

((Mesenchymal adj3 (Stem or Stroma$1 or Progenitor*)) and Cell$1).tw,ti,ab. or (Mesenchymal adj2 (Stem or Stromal or Progenitor or Multipotent or Bone marrow or Adipose or Umbilical cord or placenta*)).tw,ti,ab,mp. or "Stem Cell Transplantation".tw,ti,ab,mp. or "Stem Cells".tw,ti,ab,mp. or "Stem Cell Research".tw,ti,ab,mp. or "Cell Therapy".tw,ti,ab,mp. or "cell based therapy".tw,ti,ab,mp. or "cell-based therapy".tw,ti,ab,mp. or "Stem Cell Transplantation".tw,ti,ab,mp. or "Multipotent Stem Cells".tw,ti,ab,mp. or "Multipotent Stromal Cells".tw,ti,ab,mp. or "Mesenchymal Stem Cells".tw,ti,ab. or "Mesenchymal Stromal Cells".tw,ti,ab. or "Mesenchymal Progenitor Cells".tw,ti,ab,mp. or "Wharton Jelly Cells".tw,ti,ab,mp. or "Wharton’s Jelly Cells".tw,ti,ab,mp. or "Umbilical Cord Cell*".tw,ti,ab,mp. or "MSC".tw,ti,ab,mp. or "MSCs".tw,ti,ab. or "ADMSC".tw,ti,ab. or "ADMSCs".tw,ti,ab. or "AD-MSC".tw,ti,ab. or "AD-MSCs".tw,ti,ab,mp. or "BM-MSC".tw,ti,ab,mp. or "BM-MSCs".tw,ti,ab,mp. or "BMD-MSC".tw,ti,ab,mp. or "BMD-MSCs".tw,ti,ab,mp. or "BMDMSC".tw,ti,ab,mp. or "BMDMSCs".tw,ti,ab,mp. 418468

((Intraspinal adj2 injection$1) or (Spinal adj2 Injection$1) or (subarachnoid adj2 space$1) or (intrathecal adj2 (administration$1 or injection$1))).tw,ti,ab,mp. 27801

1 and 2 and 3 183

**Embase**

Research Strategy:

'safe':ti,ab,kw OR 'safety':ti,ab,kw OR 'side event$1':ti,ab,kw OR 'side effect$1':ti,ab,kw OR ('side':ti,ab,kw AND 'event$1':ti,ab,kw) OR ('side':ti,ab,kw AND 'effect$1':ti,ab,kw) OR 'adverse event$1':ti,ab,kw OR 'adverse effect$1':ti,ab,kw OR ('adverse':ti,ab,kw AND 'event$1':ti,ab,kw) OR ('adverse':ti,ab,kw AND 'effect$1':ti,ab,kw) OR (('adverse':ti,ab,kw OR 'undesirable':ti,ab,kw OR 'harm*':ti,ab,kw OR 'serious':ti,ab,kw OR 'toxic':ti,ab,kw) AND adj3:ti,ab,kw AND ('effect*':ti,ab,kw OR 'reaction*':ti,ab,kw OR 'event*':ti,ab,k OR 'outcome*':ti,ab,kw)) 1535832

mesenchymal:ti,ab,kw AND adj3:ti,ab,kw AND (stem:ti,ab,kw OR stroma$1:ti,ab,kw OR progenitor*:ti,ab,kw) AND cell$1:ti,ab,kw OR (mesenchymal:ti,ab,kw AND adj2:ti,ab,kw AND (((stem:ti,ab,kw OR stromal:ti,ab,kw OR progenitor:ti,ab,kw OR multipotent:ti,ab,kw OR bone:ti,ab,kw) AND marrow:ti,ab,kw OR adipose:ti,ab,kw OR umbilical:ti,ab,kw) AND cord:ti,ab,kw OR placenta*:ti,ab,kw)) OR 'stem cells':ti,ab,kw OR 'stem cell research':ti,ab,kw OR 'cell therapy':ti,ab,kw OR 'cell based therapy':ti,ab,kw OR 'cell-based therapy':ti,ab,kw OR 'stem cell transplantation':ti,ab,kw OR 'multipotent stem cells':ti,ab,kw OR 'multipotent stromal cells':ti,ab,kw OR 'mesenchymal stem cells':ti,ab,kw OR 'mesenchymal stromal cells':ti,ab,kw OR 'mesenchymal progenitor cells':ti,ab,kw OR 'wharton jelly cells':ti,ab,kw OR 'whartons jelly cells':ti,ab,kw OR 'umbilical cord cell*':ti,ab,kw OR 'msc':ti,ab,kw OR 'mscs':ti,ab,kw OR 'admsc':ti,ab,kw OR 'admscs':ti,ab,kw OR 'admsc':ti,ab,kw OR 'ad-mscs':ti,ab,kw OR 'bm-msc':ti,ab,kw OR 'bm-mscs':ti,ab,kw OR 'bmd-msc':ti,ab,kw OR 'bmd-mscs':ti,ab,kw OR 'bmdmsc':ti,ab,kw O 'bmdmscs':ti,ab,kw 441481

'injections, spinal':exp,ti,ab,kw OR 'intraspinal injections':ti,ab,kw OR 'injections, intraspinal':ti,ab,kw OR 'injection, intraspinal':ti,ab,kw OR 'intraspinal injection':ti,ab,kw OR 'spinal injections':ti,ab,kw OR 'injection, spinal':ti,ab,kw OR 'spinal injection':ti,ab,kw OR 'injections, intrathecal':ti,ab,kw OR 'injection, intrathecal':ti,ab,kw OR 'intrathecal injection':ti,ab,kw OR 'intrathecal injections':ti,ab,kw 6445

#1 AND #2 AND #3 93

**Scopus**

Research Strategy:

((TITLE-ABS-KEY("safe") OR TITLE-ABS-KEY("safety") OR TITLE-ABS-KEY("side event$1") OR TITLE-ABS-KEY("side effect$1") OR TITLE-ABS-KEY(("side" AND "event$1")) OR TITLE-ABS-KEY(("adverse" or "undesirable" or "harm*"or "serious" or "toxic") AND ("effect*" or "reaction*" or "event*" or "outcome*")))) AND ((TITLE-ABS-KEY((Mesenchymal and (Stem OR Stroma$1 OR Progenitor*) and Cell$1)) OR TITLE-ABS-KEY((Mesenchymal and (Stem OR Stromal OR Progenitor OR Multipotent OR Bone marrow OR Adipose OR Umbilical cord OR placenta*))) OR TITLE-ABS-KEY("Stem Cell Transplantation") OR TITLE-ABS-KEY("Stem Cells") OR TITLE-ABS-KEY("Stem Cell Research") OR TITLE-ABS-KEY("Cell Therapy") OR TITLE-ABS-KEY("cell based therapy") OR TITLE-ABS-KEY("cell-based therapy") OR TITLE-ABS-KEY("Stem Cell Transplantation") OR TITLE-ABS-KEY("Multipotent Stem Cells") OR TITLE-ABS-KEY("Multipotent Stromal Cells") OR TITLE-ABS-KEY("Mesenchymal Stem Cells") OR TITLE-ABS-KEY("Mesenchymal Stromal Cells") OR TITLE-ABS-KEY("Mesenchymal Progenitor Cells") OR TITLE-ABS-KEY("Wharton Jelly Cells") OR TITLE-ABS-KEY("Whartons Jelly Cells") OR TITLE-ABS-KEY("Umbilical Cord Cell*") OR TITLE-ABS-KEY("MSC") OR TITLE-ABS-KEY("MSCs") OR TITLE-ABS-KEY("ADMSC") OR TITLE-ABS-KEY("ADMSCs") OR TITLE-ABS-KEY("AD-MSC") OR TITLE-ABS-KEY("AD-MSCs") OR TITLE-ABS-KEY("BM-MSC") OR TITLE-ABS-KEY("BM-MSCs") OR TITLE-ABS-KEY("BMD-MSC") OR TITLE-ABS-KEY("BMD-MSCs") OR TITLE-ABS-KEY("BMDMSC") OR TITLE-ABS-KEY("BMDMSCs"))) AND ((TITLE-ABS-KEY("injections, spinal") OR TITLE-ABS-KEY("intraspinal injections") OR TITLE-ABS-KEY("injections, intraspinal") OR TITLE-ABS-KEY("injection, intraspinal") OR TITLE-ABS-KEY("intraspinal injection") OR TITLE-ABS-KEY("spinal injections") OR TITLE-ABS-KEY("injection, spinal") OR TITLE-ABS-KEY("spinal injection") OR TITLE-ABS-KEY("injections, intrathecal") OR TITLE-ABS-KEY("injection, intrathecal") OR TITLE-ABS-KEY("intrathecal injection") OR TITLE-ABS-KEY("intrathecal injections"))) 155 Resultados

**Cochrane Library**

Research Strategy:

("safe" or "safety" or "side event$1" or "side effect$1" or ("side" and "event$1") or ("side" and "effect$1") or "adverse event$1" or "adverse effect$1" or ("adverse" and "event$1") or ("adverse" and "effect$1") or ((adverse or undesirable or harm* or serious or toxic) adj3 (effect* or reaction* or event* or outcome*))).tw,ti,ab,mp. 599097

((Mesenchymal adj3 (Stem or Stroma$1 or Progenitor*)) and Cell$1).tw,ti,ab. or (Mesenchymal adj2 (Stem or Stromal or Progenitor or Multipotent or Bone marrow or Adipose or Umbilical cord or placenta*)).tw,ti,ab,mp. or "Stem Cell Transplantation".tw,ti,ab,mp. or "Stem Cells".tw,ti,ab,mp. or "Stem Cell Research".tw,ti,ab,mp. or "Cell Therapy".tw,ti,ab,mp. or "cell based therapy".tw,ti,ab,mp. or "cell-based therapy".tw,ti,ab,mp. or "Stem Cell Transplantation".tw,ti,ab,mp. or "Multipotent Stem Cells".tw,ti,ab,mp. or "Multipotent Stromal Cells".tw,ti,ab,mp. or "Mesenchymal Stem Cells".tw,ti,ab. or "Mesenchymal Stromal Cells".tw,ti,ab. or "Mesenchymal Progenitor Cells".tw,ti,ab,mp. or "Wharton Jelly Cells".tw,ti,ab,mp. or "Wharton’s Jelly Cells".tw,ti,ab,mp. or "Umbilical Cord Cell*".tw,ti,ab,mp. or "MSC".tw,ti,ab,mp. or "MSCs".tw,ti,ab. or "ADMSC".tw,ti,ab. or "ADMSCs".tw,ti,ab. or "AD-MSC".tw,ti,ab. or "AD-MSCs".tw,ti,ab,mp. or "BM-MSC".tw,ti,ab,mp. or "BM-MSCs".tw,ti,ab,mp. or "BMD-MSC".tw,ti,ab,mp. or "BMD-MSCs".tw,ti,ab,mp. or "BMDMSC".tw,ti,ab,mp. or "BMDMSCs".tw,ti,ab,mp. 14466

((Intraspinal adj2 injection$1) or (Spinal adj2 Injection$1) or (subarachnoid adj2 space$1) or (intrathecal adj2 (administration$1 or injection$1))).tw,ti,ab,mp. 3537

1 and 2 and 3 78

**KCI-Korean Journal Database**

Research Strategy:

((((((((((TS=("safe")) OR TS=("safety")) OR TS=("side event$1")) OR TS=("side effect$1")) OR TS=("side" AND "event$1")) OR TS=("side" AND "effect$1")) OR TS=("adverse event$1")) OR TS=("adverse effect$1")) OR TS=("adverse" AND "event$1")) OR TS=("adverse" AND "effect$1")) OR TS=((adverse or undesirable or harm* or serious or toxic) adj3 (effect* or reaction* or event* or outcome*)) Results: 63172

((((((((((((((((((((((((((((TS=((Mesenchymal adj3 (Stem OR Stroma$1 OR Progenitor*) and Cell$1))) OR TS=((Mesenchymal adj2 (Stem OR Stromal OR Progenitor OR Multipotent OR Bone marrow OR Adipose OR Umbilical cord OR placenta*)))) OR TS=("Stem Cell Transplantation")) OR TS=("Stem Cells")) OR TS=("Stem Cell Research")) OR TS=("Cell Therapy")) OR TS=("cell based therapy")) OR TS=("cell-based therapy")) OR TS=("Stem Cell Transplantation")) OR TS=("Multipotent Stem Cells")) OR TS=("Multipotent Stromal Cells")) OR TS=("Mesenchymal Stem Cells")) OR TS=("Mesenchymal Stromal Cells")) OR TS=("Mesenchymal Progenitor Cells")) OR TS=("Wharton Jelly Cells")) OR TS=("Whartons Jelly Cells")) OR TS=("Umbilical Cord Cell*")) OR TS=("MSC")) OR TS=("MSCs")) OR TS=("ADMSC")) OR TS=("ADMSCs")) OR TS=("AD-MSC")) OR TS=("AD-MSCs")) OR TS=("BM-MSC")) OR TS=("BM-MSCs")) OR TS=("BMD-MSC")) OR TS=("BMD-MSCs")) OR TS=("BMDMSC")) OR TS=("BMDMSCs") Results: 4613

(((((((((((TS=("injections, spinal")) OR TS=("intraspinal injections")) OR TS=("injections, intraspinal")) OR TS=("injection, intraspinal")) OR TS=("intraspinal injection")) OR TS=("spinal injections")) OR TS=("injection, spinal")) OR TS=("spinal injection")) OR TS=("injections, intrathecal")) OR TS=("injection, intrathecal")) OR TS=("intrathecal injection")) OR TS=("intrathecal injections") Results: 90

#1 AND #2 AND #3 Results: 2

**Web of Science Databases**

Research Strategy:

((((((((((TS=("safe")) OR TS=("safety")) OR TS=("side event$1")) OR TS=("side effect$1")) OR TS=(("side" AND "event$1"))) OR TS=(("side" AND "effect$1"))) OR TS=("adverse event$1")) OR TS=("adverse effect$1")) OR TS=(("adverse" AND "event$1"))) OR TS=(("adverse" AND "effect$1"))) OR TS=(((adverse or undesirable or harm* or serious or toxic) adj3 (effect* or reaction* or event* or outcome*))) Results: 1588470

((((((((((((((((((((((((((((TS=((Mesenchymal adj3 (Stem OR Stroma$1 OR Progenitor*) and Cell$1))) OR TS=((Mesenchymal adj2 (Stem OR Stromal OR Progenitor OR Multipotent OR Bone marrow OR Adipose OR Umbilical cord OR placenta*)))) OR TS=("Stem Cell Transplantation")) OR TS=("Stem Cells")) OR TS=("Stem Cell Research")) OR TS=("Cell Therapy")) OR TS=("cell based therapy")) OR TS=("cell-based therapy")) OR TS=("Stem Cell Transplantation")) OR TS=("Multipotent Stem Cells")) OR TS=("Multipotent Stromal Cells")) OR TS=("Mesenchymal Stem Cells")) OR TS=("Mesenchymal Stromal Cells")) OR TS=("Mesenchymal Progenitor Cells")) OR TS=("Wharton Jelly Cells")) OR TS=("Whartons Jelly Cells")) OR TS=("Umbilical Cord Cell*")) OR TS=("MSC")) OR TS=("MSCs")) OR TS=("ADMSC")) OR TS=("ADMSCs")) OR TS=("AD-MSC")) OR TS=("AD-MSCs")) OR TS=("BM-MSC")) OR TS=("BM MSCs")) OR TS=("BMD-MSC")) OR TS=("BMD-MSCs")) OR TS=("BMDMSC")) OR TS=("BMDMSCs") Results: 485246

(((((((((((TS=("injections, spinal")) OR TS=("intraspinal injections")) OR TS=("injections, intraspinal")) OR TS=("injection, intraspinal")) OR TS=("intraspinal injection")) OR TS=("spinal injections")) OR TS=("injection, spinal")) OR TS=("spinal injection")) OR TS=("injections, intrathecal")) OR TS=("injection, intrathecal")) OR TS=("intrathecal injection")) OR TS=("intrathecal injections") Results: 4428

#1 AND #2 AND #3 Results: 67

**Supplementary Table S1. MSCs Characteristics**

|  |  |  |  |  |  |  |  |  |  |
| --- | --- | --- | --- | --- | --- | --- | --- | --- | --- |
|  | **First author, year** | **Clinical population** | **Cell Type** | **Origin** | **MSCs preparation** | **Xeno-free culture medium** | **Administration frequency** | **Dosage** |  |
|  | Hlebokazov Fedor et al., 2021 | DRE | BMMSC-NTF | Autologous | Fresh | Yes | Single-dose | ≤ 10^6^ cells |  |
|  | Cudkowicz Merit E., 2022 | ALS | NurOwn® (BMMSC-NTF cells) | Autologous | Cryopreserved | Yes | Multiple-dose | 10^7^ - 10^8^ cells |  |
|  | Albu Sergiu., 2021 | SCI | UC-MSCs | Unmatched allogeneic | Cryopreserved | Yes | Single-dose | 10^7^ - 10^8^ cells |  |
|  | Song Hua., 2020 | SCI | BM-MSCs | Autologous | Fresh | Yes | Single-dose | 10^7^ - 10^8^ cells |  |
|  | Petrou Panayiota., 2020 | MS | BM-MSCs | Autologous | Cryopreserved | Yes | Multiple-dose | 10^7^ - 10^8^ cells |  |
|  | Berry James D., 2019 | ALS | BMMSC-NTF | Autologous | Fresh | Yes | Single-dose | 10^7^ - 10^8^ cells |  |
|  | Oh Ki-Wook., 2018 | ALS | BM-MSCs | Autologous | Fresh | Yes | Multiple-dose | 10^7^ - 10^8^ cells |  |
|  | Hlebokazov Fedor., 2017 | DRE | BMMSC-NTF | Autologous | Fresh | Yes | Single-dose | ≤ 10^6^ cells |  |
|  | Wang Sen., 2013 | TBI | UC-MSCs | Unmatched allogeneic | Fresh | Yes | Multiple-dose | 10^7^ - 10^8^ cells |  |
|  |  |  |  |  |  |  |  |  |  |
|  | ALS: amyotrophic lateral sclerosis; SCI: spinal cord injury; DRE: drug-resistant epilepsy; MS: multiple sclerosis; TBI: traumatic brain injury; BM-MSCs: bone marrow-derived mesenchymal stem cells; UC-MSCs: umbilical cord mesenchymal stem cells; BMMSC-NTF: bone marrow-derived mesenchymal stem cells induced to secrete high levels of neurotrophic factor; MSCs: mesenchymal stem cells; NTF: neurotrophic factor, IT: intrathecal injection. | | | | | | | |  |

**Supplementary Table S2. Univariate random effects meta-regression models**

|  |  |  |  |  |  |  |  |
| --- | --- | --- | --- | --- | --- | --- | --- |
|  | **Probability of AEs occurrence with IT MSCs therapy** | **Exp (β)** | **Standard Error** | **Lower limit** | **Upper limit** | ***p* - Values** |  |
|  |  |  |  |  |  |  |  |
|  |  |  |  |  |  |  |  |
|  | **Cell Type, BM-MSCs (base)** |  |  |  |  |  |  |
|  | BMMSC-NTF | 1.118 | 0.358 | 0.597 | 2.093 | 0.728 |  |
|  | UC-MSCs | 6.563 | 6.899 | 0.836 | 51.516 | 0.074 |  |
|  | **Origin, Autologous (base)** |  |  |  |  |  |  |
|  | Unmatched allogeneic | 6.310 | 6.414 | 0.860 | 46.273 | 0.070 |  |
|  | **MSCs preparation, Cryopreserved (base)** |  |  |  |  |  |  |
|  | Fresh | 1.426 | 0.367 | 0.861 | 2.363 | 0.168 |  |
|  | **Administration Frequency, Single dose (base)** |  |  |  |  |  |  |
|  | Multiple dose | 0.656 | 0.166 | 0.399 | 1.078 | 0.096 |  |
|  | **Dosage, ≤ 10^6^ (base)** |  |  |  |  |  |  |
|  | 10^7^ - 10^8^ cells | 0.163 | 0.175 | 0.020 | 1.342 | 0.092 |  |
|  | **Clinical Population, SCI (base)** |  |  |  |  |  |  |
|  | MS | 1.110 | 0.843 | 0.250 | 4.922 | 0.891 |  |
|  | ALS | 1.038 | 0.736 | 0.259 | 4.169 | 0.958 |  |
|  | TBI | 10.702 | 17.089 | 0.468 | 244.710 | 0.138 |  |
|  | DRE | 6.465 | 8.244 | 0.531 | 78.690 | 0.143 |  |
|  |  |  |  |  |  |  |  |
|  | * Statistical significance: p < 0·05; AEs: adverse events; MSCs: mesenchymal stem cells; MS: multiple sclerosis; ALS: amyotrophic lateral sclerosis; TBI: traumatic brain injury; SCI: spinal cord injury; IT: intrathecal; DRE: drug-resistant epilepsy | | | | | |  |

**Supplementary Table S3. Grade approach to rate the certainty from a systematic review and meta-analysis.**

|  |  |  |  |  |  |  |  |
| --- | --- | --- | --- | --- | --- | --- | --- |
|  | **Comparison** | **Main outcome measures** | **No. of studies** | **Number of participants** | **Grading of certainty of evidence** | **Downgrading due to** |  |
|  | MSCs (BM-MSC, WJ-MSC, BMMSC-NTF) vs Control group (placebo, nontreatment, standard treatment, or sham procedure) | *AEs (all studies)*: Proportion of patients with AEs in the MSCs group (433/283) and control group (243/257) | 9 | 540 | Moderate | Imprecision (serious, 1 level) |  |
|  | MSCs (BM-MSC, BMMSC-NTF) vs Control group (placebo, sham procedure, standard treatment) | *SAEs*: Proportion of patients with SAEs in the MSCs group (43/196) and control group (25/169) | 4 | 365 | Moderate | Imprecision (serious, 1 level) |  |
|  | MSCs (BM-MSC, BMMSC-NTF) vs Control group (placebo, standard treatment, or sham procedure) | *AEs (General disorders and administration site conditions)*: Proportion of patients with AEs in the MSCs group (27/135) and control group (3/108) | 4 | 243 | Moderate | Imprecision (serious, 1 level) |  |
|  | MSCs (BM-MSC, WJ-MSC, BMMSC-NTF) vs Control group (placebo, standard treatment, or sham procedure) | *AEs (Musculoskeletal and connective tissue disorders)*: Proportion of patients with AEs in the MSCs group (139/235) and control group (48/207) | 6 | 442 | High | Any study limitations were negligible, any inconsistency was negligible, and the results were precise |  |
|  | MSCs (BM-MSC, WJ-MSC, BMMSC-NTF) vs Control group (placebo, nontreatment, standard treatment, or sham procedure) | *AEs (Nervous system disorders)*: Proportion of patients with AEs in the MSCs group (99/283) and control group (59/257) | 9 | 540 | Moderate | Imprecision (serious, 1 level) |  |
|  | MSCs (BM-MSCs, BMMSC-NTF) vs Control group (placebo, standard treatment, or sham procedure) | *AEs (Infections and infestations)*: Proportion of patients with AEs in the MSCs group (6/83) and control group (3/81) | 3 | 164 | Moderate | Imprecision (serious, 1 level) |  |
|  | MSCs (BM-MSC, BMMSC-NTF) vs Control group (placebo, standard treatment) | *AEs (Respiratory, thoracic and mediastinal disorders)*: Proportion of patients with AEs in the MSCs group (7/69) and control group (2/43) | 2 | 112 | Moderate | Imprecision (serious, 1 level) |  |
|  | MSCs (BM-MSC, WJ-MSC, BMMSC-NTF) vs Control group (placebo, standard treatment) | *AEs (Gastrointestinal disorders)*: Proportion of patients with AEs in the MSCs group (33/154) and control group (20/129) | 4 | 283 | Moderate | Imprecision (serious, 1 level) |  |
|  | MSCs (BM-MSC, BMMSC-NTF) vs Control group (placebo, standard treatment, or sham procedure) | *AEs (Injury, poisoning and procedural complications)*: Proportion of patients with AEs in the MSCs group (119/160) and control group (107/157) | 3 | 317 | Moderate | Imprecision (serious, 1 level) |  |
|  | MSCs (BMMSC-NTF) vs Control group (standard treatment with AEDs) | *Numbers of responders at 12 months timepoint (seizure frequency reduced by > 50%)*. (i) BMMSC-NTF, one course (patients received a single course of cell therapy): 70% (14 out of 20 patients) vs Control group (L-): 3.8% (1 out of 26 patients). (ii) BMMSC-NTF, two courses (patients received a second course of cell therapy): 77.8% (7 out of 9 patients) vs Control group (L-): 3.8% (1 out of 26 patients). (iii) BMMSC-NTF, two courses (L+) (two courses of cell therapy combined with levetiracetam treatment): 100% (5 out of 5 patients) vs Control group (L+): 0% (0 out of 7 patients). | 1 | BM-MSCs (1 course): 20 patients; BM-MSCs (2 courses): 9 patients; BM-MSCs (2 courses (L+)): 5 patients; Control group: 33 patients. | Moderate | Imprecision (serious, 1 level) |  |
|  | MSCs (BMMSC-NTF) vs Control group (placebo) | *Efficacy results (at 28 weeks)*. (i) *≥ 1.25 points improvement in ALSFRS-R slope, n (%):* MSC-NTF: 31 (32.6%) vs Control group: 26 (27.7%); OR: 1.33; 95% CI (0.63 to 2.80); *p* = 0.45. (ii) *≥ 100 improvement in ALSFRS-R slope, n (%):* MSC-NTF: 13 (13.7%) vs Control group: 13 (13.8%); OR: 0.998; 95% CI (0.42 to 2.40); *p* = 0.997. (iii) *ALSFRS-R Total score, LS mean change from baseline (SE)*: MSC-NTF: -5.52 (0.67) vs Control group: -5.88 (0.67); LS mean difference: 0.37; 95% CI (-1.47 to 2.20); *p* = 0.69. (iv) *CAFS, LS mean at week 28 (SE)*: MSC-NTF: 73.74 (5.21) vs Control group: 72.21 (4.89); LS mean difference: 1.53; 95% CI (-10.65 to 13.72); *p* = 0.80. (v) *Slow vital capacity % predicted LS mean change from baseline (SE)*: MSC-NTF: -12.94 (1.80) vs Control group: -11.55 (1.81); LS mean difference: -1.39; 95% CI (-6.15 to 3.38), *p* = 0.56. | 1 | MSC-NTF: 95 patients; Placebo: 94 patients | High | Any study limitations were negligible, any inconsistency was negligible, and the results were precise |  |
|  | MSCs (WJ-MSCs) vs Control group (placebo) | *Clinical and neurophysiological changes*: (i) The change in pinprick sensation on the right side was significant at three months (*p* = 0.03) and reached maximum improvement at six months (*p* = 0.013), compared with baseline, following WJ-MSC infusion. (ii) *Anorectal physiology and bowel function*: Constipation (ROME III criteria): All patients presented bowel constipation, which did not change significantly after either WJ-MSC or placebo intervention (*p* > 0.05). (iii) *Severity of faecal incontinence (Wexner score)*: Patients presented low severity of faecal incontinence, which did not change significantly after interventions WJ-MSCs: 2.88 ± 1.5 vs Placebo: 4.75 ± 1.1 (Effect size: 0.12; *p* > 0.05). (iv) *Urinary-specific quality of life (Qualiven questionnaire)*: There were no significant changes in the impact of urinary problems on quality of life following WJ-MSCs or placebo. | 1 | WJ-MSCs: 5 patients; Placebo: 5 patients | High | Any study limitations were negligible, any inconsistency was negligible, and the results were precise |  |
|  | MSCs (BM-MSCs) vs Control group (decompression + internal fixation + conventional medical treatments) | (i) *Comparison of the total effective rate between the two groups (at 12 months)*: The total effective rate in the experimental group was 94.4% (17/18 patients), which was higher than that of the control group 11/18 patients (61.1%; *p* = 0.045). (ii) *Comparison of neurological recovery between the two groups (at 12 months): SCIM-III score:* BM-MSCs group (mean 72.53 ± sd 4.31) vs Control group (mean 63.52 ± sd 5.04); *p* < 0.05. (iii) *ASIA exercise score*: BM-MSCs group (mean 81.13 ± sd 3.81) vs Control group (mean 70.89 ± sd 4.77); *p* < 0.05. (iv) *Botsford score*: BM-MSCs group (mean 22.45 ± sd 2.13) vs Control group (mean 18.07 ± sd 2.32); *p* < 0.05. All scores were higher than before treatment in both groups, and the increase in the experimental group was significantly higher than that in the control group (all *p* < 0.05). | 1 | BM-MSCs: 18 patients; Control group: 18 patients | Moderate | Imprecision (serious, 1 level) |  |
|  | MSCs (BM-MSCs) vs Control group (sham treatment) | *Clinical efficacy (at six months).* (i) *Treatment failure (Increase in at least one Functional System score):* MB-MSCs 31.0% (9/29) vs Control group 76.7% (23/30); *p* = 0.0004. (ii) *Change in EDSS; median (IQR):* MB-MSCs (–0.20 ± 0.3) vs Control group (+0.30 ± 0.4); *p* < 0.0001. (iii) *Relapses per patient, mean ± sd:* MB-MSCs (0.06 ± 0.25) vs Control group (0.28 ± 0.58); *p* = 0.0005. (iv) *Proportion/patients relapse-free (n):* MB-MSCs 93.8% (30/32) vs Control group 78.1% (25/32); *p* = 0.001. | 1 | BM-MSCs: 32 patients; Control group: 32 patients | High | Any study limitations were negligible, any inconsistency was negligible, and the results were precise |  |
|  | MSCs (MSC-NTF) vs Control group (placebo) | *ALSFRS-R LS mean slope change.* (i) *Change in the ALSFRS-R slope post-treatment:* MSC-NTF (+1.7 points/month) vs Control group (- 0.4 points/month) at two weeks; *p* = 0.110; MSC-NTF (+ 0.6 points/month) vs Control group (- 0.03 points/month) at four weeks; p = 0.368; After eight weeks, the change in slope was similar in both treatment arms. (ii) *Comparison of LS means of the post-transplant ALSFRS-R slope minus the pretransplantation slope:* MSC-NTF (+ 3.3 points/month) vs Control group (- 1.3 points/month) at two weeks, p = 0.021; MSC-NTF (+ 2.0 points/month) vs Control group (- 0.1 points/month) at four weeks, p = 0.033; and a continued trend for improvement in the MSC-NTF group at all remaining time points. *ALSFRS-R responder analyses.* (iii) *≥ 1.5 points/month improvement in ALSFRS-R slope post-treatment compared to pretreatment at week 4:* MSC-NTF 47% vs Control group 9%; *p* = 0.033. A higher proportion of responders was observed in the MSC-NTF group compared to the control group at all time points. (iv) *≥ 1.5 points/month improvement in ALSFRS-R slope of MSC-NTF group compared to the Control group:* MSC-NTF 80% vs Control group 0%; 95% CI of difference of proportions (59.8% to 100%); *p* = 0.004 (at week 4). MSC-NTF 53% vs Control group 0%; 95% CI of difference of proportions (28.1% to 78.6%; *p* = 0.046 (at week 8). (v) *Slow vital capacity:* No significant treatment effects were noted in the rapid progressors group. (vi) *Hand-held dynamometry* did not demonstrate significant side-to-side difference in HHD muscle strength score slopes over 24 weeks. | 1 | MSC-NTF: 36 patients; Control group: 12 patients | High | Any study limitations were negligible, any inconsistency was negligible, and the results were precise |  |
|  | MSCs (BM-MSCs) vs Control group (riluzole treatment - 100mg/day) | (i) *ALSFRS-R score change from baseline to 6 months, mean difference between groups:* 3.38 (SE: 1.07; 95% CI 1.23 to 5.54; *p* = 0.003). (ii) *Changes in ALSFRS-R slope between lead-in and follow-up period, up to 6 months:* 0.92 (SE: 0.38; 95% CI 0.16 to 1.67; *p* = 0.018). (iii) *AALS score change from baseline to 4 months:* -7.18 (SE: 2.76; 95% CI -12.71 to -1.64; *p* = 0.009). (iv) *Changes in AALS slope between lead-in and follow-up period:* -1.80 (SE: 1.14; 95% CI - 4.08 to 0.48; *p* = 0.119). (v) *SF-36 change from baseline to 4 months:* 2.78 (SE: 12.18; 95% CI -3.74 to 9.29; *p* = 0.397). *Responder Analysis:* (i) *≥ 50% improvement in ALSFRS-R slope, good responders, n (%):* BM-MSCs group: 20 (63%) vs Control group: 6 (22%); *p* = 0.002 (at six months). (ii) *≥ 75% improvement in ALSFRS-R slope, n (%):* BM-MSCs group: 17 (53%) vs Control group: 3 (11%); *p* = 0.001 (at six months). (iii) *100% improvement in ALSFRS-R slope, n (%):* BM-MSCs group: 8 (25%) vs Control group: 1 (4%); p = 0.031 (at six months). | 1 | BM-MSC: 31 patients; Control group: 25 patients | Moderate | Imprecision (serious, 1 level) |  |
|  | AEDs + MSCs (MSC-NTF) vs Control group (AED) | *Number of responders to treatment in AED + MSC-NTF group and Control group (at endpoint):* MSC-NTF group: 8/10 (80%) vs Control group: 2/12 (16.7%). The patients in the cell therapy group showed better results; however, only the seizure frequency parameter has achieved statistical significance. | 1 | MSC-NTF: 10 patients; Control group: 12 patients | Moderate | Imprecision (serious, 1 level) |  |
|  | UC-MSCs group vs Control group | *Total scale scores of the FMA. Delta value (difference) of FMA scores:* UC-MSC group: 5.60 ± 3.15 vs Control group: 0.25 ± 0.64; *p* < 0.05. *Total scale scores of the FIM. Delta value (difference) of FIM scores:* UC-MSC group: 4.20 ± 3.58 vs Control group: 0.65 ± 1.42; p < 0.05. | 1 | UC-MSCs group | Moderate | Imprecision (serious, 1 level) |  |
|  |  | | | | | |  |
|  | AEs: adverse events; SAEs: serious adverse events; AEDs: anti-epileptic drugs; (L-) without levetiracetam; (L+) with levetiracetam; EDSS: expanded disability status scale; IQR: interquartile range; ALS: amyotrophic lateral sclerosis; ALSFRS-R: revised ALS functional rating scale; CAFS: combined analysis of function and survival; AALS: appel ALS rating scale; FMA: Fugl-Meyer assessments; FIM: functional independence measures; BM-MSC: bone marrow-derived mesenchymal stem cells; WJ-MSC: Wharton jelly mesenchymal stem cells; BMMSC-NTF: bone marrow-derived mesenchymal stem cells induced to secrete high levels of neurotrophic factor; MSCs: mesenchymal stem cells; NTF: neurotrophic factor; SCIM-III: independent scale of spinal cord injury III; HHD: hand-held dynamometry; LS: least-square; CI: confidence interval; SD: standard deviation. | | | | | |  |
|  |  |  |  |  |  |  |  |
|  |  |  |  |  |  |  |  |
|  |  |  |  |  |  |  |  |

**Supplementary Table S4. Summary of active intrathecal MSCs-based clinical trials that included a control group.**

|  |  |  |  |  |  |  |  |  |  |  |  |  |  |
| --- | --- | --- | --- | --- | --- | --- | --- | --- | --- | --- | --- | --- | --- |
|  | **Clinical Trial Identifier** | **Registration Year** | **Location Countries** | **Phase** | **Recruitment Status** | **Official Title** | **Condition** | **Intervention** | **Control** | **Outcome Measures** | **Study Sponsor** | **Estimated Study Completion Date** |  |
|  | NCT05698017 | 2023 | Israel | Phase I/IIa | Recruiting participants | Phase I/IIa randomised controlled study for treatment of early- to moderate stage multiple system atrophy patients with the investigational allogeneic cell therapy product (hOMSC300) | MSA | Human oral mucosa stem cells (hOMSC300) | Sham procedure (Lumbar puncture only) | Treatment-related AEs and SAEs per dose group | Cytora Ltd. | February, 2026 |  |
|  | NCT04749667 | 2021 | Norway | Phase I/II | Recruiting participants | Study of mesenchymal autologous stem cells as regenerative treatment for multiple sclerosis | MS | Autologous bone-marrow-derived MSCs | Isotonic saline | Rate and nature of AEs, SAEs and preliminary efficacy profile | Haukeland University Hospital | January, 2025 |  |
|  | NCT05532943 | 2022 | Taiwan | Phase I/IIa | Recruiting participants | A Seamless phase I/IIa clinical study to evaluate the safety and efficacy of allogeneic umbilical cord mesenchymal stem cells in patients with multiple sclerosis | MS | Allogeneic UC-MSCs will be IV infusion followed by IT infusion | Normal saline will be IV infusion followed by sham-IT infusion | SAEs and AEs incidences over the study period | Ever Supreme Bio Technology Co., Ltd. | December, 2026 |  |
|  | NCT03378414 | 2023 | China | Phase II | Not yet recruiting | Clinical research on the safety/efficacy of umbilical cord mesenchymal stem cells therapy for patients with Spinocerebellar ataxia | SCA | IV infusion and IT injection of UC-MSCs | Control group: No intervention | Safety profile and preliminary efficacy profile | Sclnow Biotechnology Co., Ltd. | December 2024 |  |
|  | NCT03521323 | 2019 | China | Phase II | Recruiting participants | The Effect of intrathecal transplantation of umbilical cord mesenchymal stem cells in patients with early stage of chronic spinal cord injury: A multicenter, randomised, controlled trial | SCI | IT transplantation of UC-MSC | Placebos: saline, sham operation | Safety profile and preliminary efficacy profile | Third affiliated hospital, Sun Yat-Sen University | Unknown |  |
|  | NCT05671796 | 2023 | Brazil | Phase II | Not yet recruiting | Phase 2, double-blind, placebo-controlled, randomised clinical trial of autologous bone marrow stem cell transplantation in patients with subacute spinal cord injury | SCI | Two IT autologous bone marrow stem cell transplantation | Placebo: two subcutaneous injections of 1 ml each, containing glycophysiological solution | Safety profile and preliminary efficacy profile | SENAI CIMATEC | December, 2023 |  |
|  | NCT03935724 | 2019 | Denmark and Spain | Phase II/III | Recruiting participants | A Multi-center, double-blind, randomised, placebo-controlled, delayed start phase II/III study to assess the efficacy and safety of neuro-cells in (sub)acute spinal cord injury patients | SCI | IT intervention with neuro-cells (autologous fresh stem cells) treatment on days 1-2 | Placebo treatment on days 1-2 | Safety profile and Efficacy profile | Neuroplast BV | June, 2024 |  |
|  | NCT05292625 | 2022 | Vietnam | Phase I/II | Recruiting participants | Outcomes of umbilical cord blood-derived mesenchymal stem cell (UC-MSC) infusion in patients with neurological complications after ischemic stroke | IS | UC-MSC infusion IV and IT route | Control group: standard stroke treatment and rehabilitation therapy | AEs and SAEs | Vinmec Research Institute of Stem Cell and Gene Technology | June, 2023 |  |
|  | NCT04520373 | 2020 | United States | Phase II | Recruiting participants | CELLTOP part II: A phase II clinical trial of autologous adipose-derived mesenchymal stem cells in the treatment of paralysis due to traumatic spinal cord injury | SCI | Autologous, AD-MSCs | Control group: occupational and physical therapy | Correlation of AEs to study drug | Mayo Clinic | June 2024 |  |
|  | EudraCT Number 2021-000346-18 | 2021 | Spain | Phase I/II | Recruiting participants | A phase I/II, randomised, double-blind, placebo-controlled, parallel, 2-arms clinical trial to assess the safety and efficacy of intrathecal administration of WJ-MSC in chronic traumatic cervical incomplete spinal cord injury | SCI | Ex vivo cultured human MSCs from Wharton jelly (XCEL-UMC-BETA) | Placebo: infusion via IT | To assess the safety of MSCs from Wharton Jelly (XCEL-UMC-BETA) infusion via IT | Banc de Sang i Teixits | Not Provided |  |
|  |  |  |  |  |  |  |  |  |  |  |  |  |  |
|  | AEs: adverse events; SAEs: serious adverse events; IV: intravenous; IT: intrathecal; MSCs: mesenchymal stem cells; UC-MSCs: umbilical cord mesenchymal stem cell; AD-MSCs: adipose-derived mesenchymal stem cells; MSA: multiple system atrophy; MS: multiple sclerosis; SCI: spinal cord injury; IS: Ischemic stroke; SCA: Spinocerebellar ataxia. | | | | | | | | | | | |  |
